# Olfactomedin-4 improves cutaneous wound healing by promoting skin cell proliferation and migration through POU5F1/OCT4 and ESR1 signalling cascades

**DOI:** 10.1007/s00018-022-04202-8

**Published:** 2022-02-26

**Authors:** Mariliis Klaas, Kristina Mäemets-Allas, Elizabeth Heinmäe, Heli Lagus, Terje Arak, Mart Eller, Külli Kingo, Esko Kankuri, Viljar Jaks

**Affiliations:** 1 Institute of Molecular and Cell Biology, University of Tartu, Riia 23b, 51010 Tartu, Estonia; 2 Department of Plastic Surgery and Wound Healing Centre, Helsinki University Hospital, University of Helsinki, Helsinki, Finland; 3 Surgery Clinic, Tartu University Hospital, Puusepa 8, 50406 Tartu, Estonia; 4 Dermatology Clinic, Tartu University Hospital, Raja 31, 50417 Tartu, Estonia; 5 Department of Pharmacology, Faculty of Medicine, University of Helsinki, Helsinki, Finland

**Keywords:** Skin regeneration, Wound healing, Skin burns, Olfactomedin-4, Keratinocytes, Psoriasis

## Abstract

Olfactomedin-4 (OLFM4) is an olfactomedin-domain-containing glycoprotein, which regulates cell adhesion, proliferation, gastrointestinal inflammation, innate immunity and cancer metastasis. In the present study we investigated its role in skin regeneration. We found that OLFM4 expression is transiently upregulated in the proliferative phase of cutaneous wound healing in humans as well as in mice. Moreover, a significant increase in OLFM4 expression was detected in the skin of lesional psoriasis, a chronic inflammatory disease characterized by keratinocyte hyperproliferation. In vitro experiments demonstrated that OLFM4 selectively stimulated keratinocyte proliferation and increased both keratinocyte and fibroblast migration. Using proteotranscriptomic pathway analysis we revealed that transcription factors POU5F1/OCT4 and ESR1 acted as hubs for OLFM4-induced signalling in keratinocytes. In vivo experiments utilizing mouse splinted full-thickness cutaneous wound healing model showed that application of recombinant OLFM4 protein can significantly improve wound healing efficacy. Taken together, our results suggest that OLFM4 acts as a transiently upregulated inflammatory signal that promotes wound healing by regulating both dermal and epidermal cell compartments of the skin.

**Supplementary Information:**

The online version contains supplementary material available at 10.1007/s00018-022-04202-8.

## Introduction

Mammalian skin is designed to act as a barrier to the environment, providing mechanical, ultraviolet light and chemical protection, preventing pathogen invasion and microbial growth, participating in thermal regulation, fluid homeostasis and contributing to the production of vital hormones. Cutaneous wound healing is a highly conserved but complex process that is designed to restore efficiently its functions and protect the homeostasis of the organism, while the integrity of the skin is lost.

Wound healing involves a series of tightly orchestrated spatially and temporally overlapping processes including blood coagulation, inflammation, keratinocyte migration and proliferation as well as tissue and extracellular matrix (ECM) remodelling [1]. A variety of cell types and response modulators, such as growth factors, cytokines, matrix metalloproteinases, cellular receptors, and extracellular matrix components are involved in initiation and proper progression of wound healing. As demonstrated recently by us and others [2, 3], coordinated signals from the ECM and from the mesenchymal and infiltrated cells instigate and promote the stepwise progression of wound healing towards re-epithelialization and scar maturation.

The course of wound healing is strongly influenced by the migration and proliferation activity of the cells at the site of injury. Olfactomedin 4 (OLFM4), a secreted glycoprotein of the olfactomedin family, has been shown to be an important regulator of cell adhesion and migration during tissue regeneration [4, 5]. Previously, we demonstrated that Olfm4 specifically stimulated hepatocyte proliferation in vitro, indicating that Olfm4 upregulation may be required to sustain the proliferation of hepatocytes in injured livers [5]. OLFM4 is highly expressed in Lgr5 + pluripotent stem cells of the intestinal crypts, and it is, therefore, considered as a robust stem cell marker [6]. Under physiological conditions, high expression of OLFM4 is detected in small intestine, colon and prostate, while moderate OLMF4 expression is found in the stomach and bone marrow [7, 8]. In line with its potential role in stem cell maintenance the OLFM4 overexpression has been found in various types of malignancies and hyperproliferative processes [8, 9], including gastric cancer [10], pancreatic, colon, lung and breast cancer [11], colorectal adenomas and liver metastases of colorectal origin [12], endometriosis [13], and cervical cancer [9]. However, in poorly differentiated, advanced-stage metastatic tumors OLFM4 levels were decreased [14]. Infection and inflammation have been shown to increase the expression of OLFM4, and its high expression has been reported in gastric biopsies of patients with *Helicobacter pylori* infection and inflammatory bowel disease [15]. These results indicate a dynamic role for OLFM4 in tissue homeostasis and suggest that OLFM4 has time- and concentration-dependent roles in inflammatory pathologies [16].

As the processes involved in wound healing (cell migration, proliferation, tissue homeostasis, inflammation, etc.) overlap with those regulated by OLFM4, we, for the first time, studied the role of OLFM4 in cutaneous wound healing. We found that the expression of OLFM4 was increased in human skin graft-treated burn wounds, in lesions of a chronic inflammatory dermatosis—psoriasis—and during skin wound healing in mice. The presence of recombinant OLFM4 improved the migration and proliferation of primary keratinocytes in vitro and enhanced the wound healing in vivo. Proteotranscriptomic analysis suggested that OLFM4 elicits its effects via POU5F1/OCT4 and ESR1 signalling, which was predicted to upregulate several migration-promoting factors, and downregulated the expression of PTEN. Our results suggest that OLFM4 acts as a molecular switch for cell migration and proliferation during cutaneous wound healing.

## Materials and methods

### Mouse wound healing experiments

Male 8-week C57/BL6 mice were used for initial wound healing experiments. Due to an acute inflammatory reaction towards recombinant human OLFM4 in C57/BL6 mice the immunodeficient female 8-week-old BALB/c-nu/nu mice were used for the wound healing assay that involved this protein. General anaesthesia was induced using 2–3% isoflurane in 100% oxygen (flow rate 1 L/min) and was maintained using 1% isoflurane. A 6-mm biopsy punch (Kai Medical, Solingen, Germany) was used to create full-thickness dermal wounds in the dorsal skin of mice; the removed skin samples were considered as day-0 healthy skin controls. For the splinted wound healing model two wounds were introduced into the dorsal skin of each mouse. To avoid wound contraction, a silicone splint (2–3 mm wide, inner diameter 6 mm) cut from a 0.5 mm thick silicone sheet (Grace Bio-Labs, Bend, OR, USA) was applied around the wound and fixed using a cyanoacrylate adhesive and surgical stitches. The wounds were covered with a transparent occlusive dressing (Tegaderm, 3 M, Maplewood, MN, USA). For testing the effect of OLFM4 protein, 1 µg of purified recombinant human OLFM4 protein (R&D Systems, Minneapolis, MN, USA, product code 10261-OL-050) dissolved in 10 µl of phosphate buffered saline (PBS) was applied daily to the wounds. Equal volumes of PBS were applied to the wounds of the animals in the control group. The occlusive dressings were replaced after each application round. Wounds were measured daily throughout the experiment. The mice were sacrificed at 0-, 2-, 4-, 6-, 8- or 12-day timepoints and the skin samples from the healing wounds were embedded in O.C.T compound (Tissue-Tek, Sakura Finetek Europe B.V., Alphen aan den Rijn, the Netherlands) and stored at − 80 °C until further analysis. 10-μm-thick frozen sections were cut for immunofluorescence and histological analysis. All procedures involving animals were conducted according to the guidelines approved by the Commission of Laboratory Animal License at the Estonian Ministry of Agriculture (license no 180).

### Burn injury samples

The study involving burn injury patients was conducted according to Declaration of Helsinki principles and was approved by the Research Ethics Committee of the Helsinki University Hospital (DNro 101/E6/2000). Informed consent was obtained from all participants. Samples from ten patients (age range 19–58 years) with large (total burn surface area range 22–45%) deep third-degree burns were collected from the study area at 3, 14 and 21 days after the wound excision with 3 mm biopsy punches for immunofluorescence analysis. Healthy normal skin from healthy volunteers was collected from similar locations that were not exposed to the sun. Formalin-fixed samples were embedded in paraffin and paraffin-embedded tissue blocks were cut on microtome into 4.5-µm-thick sections which were mounted on Superfrost Plus slides (Thermo Scientific, Braunschweig, Germany). Deparaffinization and heat-induced antigen retrieval in 10 mM sodium citrate buffer, pH 6.0, was performed before immunofluorescence analysis.

### Psoriasis samples

Adult patients with plaque psoriasis were recruited from the Tartu University Hospital at the Clinic of Dermatology between 2013 and 2015. This collection of tissue samples was approved by the Research Ethics Committee of the University of Tartu (permission number 245/M-18). The Declaration of Helsinki protocols were followed, and informed written consent was obtained from all participants. 3-mm punch biopsies were taken from the well-defined psoriatic lesional skin from the upper arm and torso of psoriasis patients and healthy volunteers from similar locations of the skin that was not exposed to the sun. Tissue samples were embedded in O.C.T compound (Tissue-Tek, Sakura Finetek Europe B.V., Alphen aan den Rijn, the Netherlands) and stored at − 80 °C for further analysis. For immunofluorescence 10-μm-thick frozen sections were cut.

### Immunofluorescence analysis

Tissue sections and cells grown on coverslips were fixed with 4% paraformaldehyde and permeabilized with 0.2% Triton X-100. After blocking with 5% normal donkey serum (Sigma-Aldrich, Merck Group, Darmstadt, Germany), the samples were incubated with primary antibodies overnight at + 4 °C, followed by incubation with fluorochrome-conjugated secondary antibodies. Used antibodies are listed in Supplementary Table S1. Nuclei were counterstained with DAPI (0.1 µg/ml, Thermo Fisher Scientific, Eugene, OR, USA). Images were captured with Olympus IX81 CellR microscope (Olympus Corporation, Hamburg, Germany) equipped with Hamamatsu Orca ER (Hamamatsu Photonics, Herrsching am Ammersee, Germany) camera and 10 × or 40 × objective and processed using Hokawo 2.1 software (Hamamatsu Photonics). The fluorescence intensity was quantified using ImageJ software [17]. For quantifying the fluorescence intensities, the areas of the analysed regions were measured and the fluorescence intensity of the analysed region was normalized to its area. Same morphological criteria were followed when manually delineating the areas for analysis across all samples to avoid bias. For cells grown on coverslips, the level of CTCF (Corrected total cell fluorescence) was calculated using the following formula: CTCF = Integrated Density − (Area of analyzed cell × Mean fluorescence of background readings). The fluorescence of 80–110 cells in each sample was quantified.

### Keratinocyte culture

Skin from healthy donors (collected from breast reduction surgeries, Tartu University Clinics; ethics approval 292/T-4) was cut into small pieces and incubated in 2.4 U/ml dispase (Thermo Fisher Scientific, Grand Island, NY, USA) solution overnight at 4 °C. Epidermis was separated and further dissociated using 0.05% trypsin solution (Thermo Fisher Scientific). The separated keratinocytes were cultured in Keratinocyte-SFM medium containing 50 µg/ml bovine pituitary extract, 0.5 ng/ml recombinant epidermal growth factor and 10 ng/ml recombinant keratinocyte growth factor (all from Thermo Fisher Scientific, Grand Island, NY, USA) and antibiotic–antimycotic solution (Gibco, Thermo Fisher Scientific) with a final concentration of 100 U/ml penicillin, 100 µg/ml streptomycin and 0.25 µg/ml amphotericin B. Pooled keratinocytes from 3 donors at passages 2–4 were used in experiments. Keratinocytes were cultured on tissue culture dishes (Corning Incorporated, Corning, NY, USA) that were pre-coated with collagen type I solution (Thermo Fisher Scientific) at a concentration of 5 μg/cm^2^, according to the manufacturer’s instructions for thin coating procedure.

### Fibroblast culture

Human primary fibroblast cultures were established by explant culture method. Briefly, skin from healthy donors (collected from breast reduction surgeries, Tartu University Clinics; ethics approval 292/T-4) was cut into small pieces and adhered to tissue culture dishes (Corning Incorporated). Skin pieces were covered in medium, that contained DMEM medium supplemented with 10% (v/v) fetal bovine serum (both Gibco, Thermo Fisher Scientific) and penicillin–streptomycin solution (Gibco, Thermo Fisher Scientific) resulting a final concentration of 100 U/ml penicillin and 100 µg/ml streptomycin. Cells were allowed to migrate from dermis for 10–14 days. Cells from at least 3 patients were pooled and stored as frozen stocks for further experiments.

### Scratch assay

Keratinocytes or fibroblasts were seeded into wells of a 24-well plate (Corning Incorporated) and cultured until nearly confluent. Cells were stimulated with 1 µg/ml recombinant OLFM4 (R&D Systems) or control medium (regular growth medium containing vehicle PBS), respectively, for 24 h prior the scratch assay. Next, the cells were incubated with the DNA synthesis inhibitor mitomycin C (Sigma-Aldrich) at final concentration 5 μg/ml for 3 h and scratches were introduced to the cell monolayers using a sterile 1 ml pipette tip. The scratches were imaged under the Nikon Eclipse TS100 microscope (Nikon Instruments, Melville, NY, USA) and the photographs were captured using a digital camera head (DS-Vi1, Nikon) equipped with a stand-alone controller and display unit (DS-L3, Nikon). Images were captured immediately after creating the wounds (timepoint 0) and every 3 or 6 h thereafter. The wound width was measured from the images using ImageJ software [17] and the wound closure percentages were calculated.

### Transwell migration assay

Before the start of the migration assay, the keratinocytes or fibroblasts were stimulated with 1 µg/ml recombinant OLFM4 (R&D Systems) or control medium (regular growth medium containing vehicle PBS) for 24 h and treated with 5 μg/ml mitomycin C (Sigma-Aldrich) for 3 h. A total of 1 × 10^5^ keratinocytes or 1.5 × 10^4^ fibroblasts were seeded in serum-free medium on the upper membrane of the transwell chamber (6.5 mm transwell with 8.0 µm pore polycarbonate membrane insert, Corning Incorporated, Kennebunk, ME, USA). Cells were allowed to migrate for 24 h and then fixed and stained with 0.5% Coomassie Brilliant Blue G-250 (Sigma-Aldrich). The cells that did not migrate through the membrane were removed with a moist cotton swab. Pictures were taken from 6 different places of each well under 10 × objective lens mounted on Nikon Eclipse TS100 microscope (Nikon Instruments) equipped with a digital camera head (DS-Vi1, Nikon) and a stand-alone controller and display unit (DS-L3, Nikon). The cells that migrated through the membrane were quantified per each field of view.

### RNA isolation

70–80% confluent keratinocytes and fibroblasts grown on 60-mm-diameter cell culture dishes (Corning Incorporated) were stimulated with 1 µg/ml recombinant OLFM4 (R&D Systems) or control medium (regular growth medium containing vehicle PBS) for 4 h. Total RNA was separated using the RNeasy Mini Kit (Qiagen, Hilden, Germany) according to the manufacturer’s instructions and processed for RNA-sequencing or cDNA synthesis and RT-qPCR. The quality and integrity of separated RNA were determined using Agilent 2200 TapeStation system. All samples processed for RNA-sequencing had RIN^e^ values 9.9 or greater.

### RNA-sequencing analysis

RNA-sequencing analysis and read mapping was performed as a service at EMBL Genomics Core Facility (Heidelberg, Germany). Individually barcoded stranded mRNA-seq libraries were prepared from high quality total RNA samples (~ 200 ng/sample) using the New England Biolabs NEBNext RNA Ultra II Directional Kit implemented on the liquid handling Beckman Coulter i7 dual hybrid workstation. Obtained libraries that passed the QC step were pooled in equimolar amounts; 2 pM solution of this pool was loaded on the Illumina sequencer NextSeq 500 and sequenced uni-directionally, generating ~ 500 million reads, each 85 bases long. The Galaxy platform (https://usegalaxy.eu/) [18] was used for transcriptomics analysis. RNA STAR (Galaxy Version 2.7.8a) was used for sequence alignment to reference genome GRCh38/h38.87 and featureCounts (Galaxy Version 2.0.1) was used to count the number of reads. Differential expression analysis was conducted using the DESeq2 package [19]. Data was uploaded to Gene Expression Omnibus (https://www.ncbi.nlm.nih.gov/geo/) (GEO, accession no. GSE188919). A gene was considered to be differentially expressed if the adjusted *p* < 0.05.

### cDNA synthesis and RT-qPCR

Total RNA was reverse transcribed with a RevertAid First Strand cDNA Synthesis Kit (Thermo Scientific) according to manufacturer’s instructions. qPCR was performed using Maxima SYBR Green/ROX qPCR Master Mix reagents (Thermo Fisher Scientific) with following primers: *SOX-11*-fw, 5′ GGAGAGCTTGGAAGCGGAGA 3′ and *SOX-11*-rev 5′ CAAGCCATGAATTCGCCCTC 3′, *HPRT1*-fw 5′ CCCTGGCGTCGTGATTAGTG 3′ and *HPRT1* -rev 5′ GTGATGGCCTCCCATCTCCT 3′. All qPCR reactions were carried out with a LightCycler^®^ 480 Instrument II (Roche) and the data acquired were analyzed with LightCycler^®^ 480 Software (Roche). Target genes mRNA expression was normalized to endogenous mRNA level of the housekeeping reference gene *HPRT1*. The qPCR reactions were performed at least 3 times for each sample.

### Proteomics analysis

Keratinocytes were grown on 100-mm-diameter cell culture dishes (Falcon Corning, Corning) until 70–80% confluency and then stimulated with 4 ml regular growth medium containing 1 µg/ml recombinant OLFM4 (R&D Systems) or control medium (regular growth medium containing vehicle PBS) for 24 h. Cells were lysed in a NP40 buffer containing protease inhibitors (Halt Protease Inhibitor Cocktail, Thermo Fisher Scientific, Rockford, IL, USA). For the full proteome analysis, 5 µg of protein was precipitated with 100% (w/v) Trichoroacetic Acid/Sodium Deoxycholate solution. Next, proteins were reduced, alkylated and digested by Lys-C protease and trypsin. Peptides were analysed on an Ultimate 3500 RSLCnano (Dionex, Sunnyvale, CA, USA) nano-LC system with a C18 cartridge column (Dionex) and an in-house packed (3 µm ReproSil-Pur C18AQ particles, Dr. Maisch GmbH, Ammerbuch, Germany) 50 cm 75 µm ID emitter-columns (New Objective) using a 120 min 8–40% B gradient, where buffer A was 0.1% formic acid in water and B, 0.1% formic acid in 80% acetonitrile. Elution was performed at 250 nl/min (spray voltage 2.4 kV) to a Q Exactive HF (Thermo Fisher Scientific) mass-spectrometer operating with a top-20 MS/MS strategy. One full MS 350–1400 m/z scan at a resolution setting of 60,000 (injection time 50 ms, ion target 3e6 ions) was followed by 20 MS/MS scans at a resolution of 15,000 (15 ms, 1e5 ions). Dynamic exclusion was set to 40 s. Only charge states + 2 to + 5 were analyzed.

Raw data were identified and quantified with MaxQuant 1.6.15.0 software package. Search was performed against UniProt (www.uniprot.org) human database using the tryptic digestion rule. Methionine oxidation and protein N-terminal acetylation were set as variable modifications, while cysteine carbamidomethylation was defined as a fixed modification. Only identifications with at least 1 peptide = 7 amino acids long (with up to 2 missed cleavages) were accepted and transfer of identifications between runs based on accurate mass and retention time was enabled. Label-free normalization with MaxQuant LFQ algorithm was also applied. LFQ ratio count (i.e., number of quantified peptides for reporting a protein intensity) was set to 1. Peptide-spectrum match and protein false discovery rate (FDR) was kept below 1% using a target-decoy approach. The mass spectrometry proteomics data are available via ProteomeXchange with identifier PXD029881.

### Ingenuity pathway analysis

Pathway analyses were performed using similar workflow as reported earlier [20] with minor alterations. Briefly, differentially expressed gene (DEG) and protein (DEP) data were imported into IPA Ingenuity Pathway Analysis software (Qiagen, Version 62089861). To provide an unsupervised initial analysis of pathways and signalling routes activated by OLFM4, we first evaluated the expression of genes and proteins with more than 1.5-fold increases and *p* < 0.3. After the unsupervised first analysis, these criteria were increasingly tightened to achieve final cutoff values for DEGs (|log2 fold change|> 1, *p* < 0.1) and DEPs (|log2 fold change|> 0.6, *p* < 0.1) in the multiomic analysis. The list of DEGs was then used to connect a pathway network utilizing the inbuilt feature of IPA for connecting gene expression node interactions with both direct and indirect connections, all confidence levels, and from several database sources; including interactions from BioGrid, Biomolecular Interaction Network Database (BIND), Cognia, Database of Interacting Proteins (DIP), Mammalian Protein–Protein Interaction Database (MIPS) and Ingenuity Expert Findings. For identifying the central nodes in the resulting interconnected gene expression pathway, the hub node integrating this interaction network in radial view was selected with the most upregulated transcription factor to build the OLFM4-induced integrating proteotranscriptomics network. Connections from these initiator nodes were expanded using the same IPA's inbuilt cross-database-integrating feature by targeting the interaction search in IPA solely to differentially expressed genes (DEGs) and differentially expressed proteins (DEPs) identified in our study. Due to the less conservative *p* value for significance, specific validations were carried out using qRT-PCR and results are presented with both log2 fold change values as well as *p* values. Moreover, the canonical pathways identified in IPA to be significantly activated and the DEGs and DEPs contributing to those pathways were selected for further network analysis. After retrieving an IPA-generated list of the significantly activated pathways and their DEGs and DEPs, we carried out GeneOntology (GO)-supervised evaluation of DEGs and DEPs using the final cutoff values to retrieve the heatmaps. Proteotranscriptomic activation analysis of DEGs with more than 2 log2 fold change and DEPs with more than 1 log2 fold change increased expression was carried out to provide a multiomic network analysis. This analysis was carried out using String 11.5 interaction association network discovery tools [21]. The DEGs and DEPs were imported into String 11.5 and a network using a minimum interaction score of 0.15 was constructed. For visualization the String network was imported into Cytoscape 3.9.0 [22]. Visualization used Nested network style and yFiles Organic layout under academic licensing ([23]; http://www.yworks.com). Final graphical layout designs of the networks were then carried out in Inkscape (1.0.1.; https://inkscape.org).

### Data analysis and statistics

Statistical significance was determined by one-way ANOVA followed by Dunnett’s post-test (multiple comparisons) or Student’s *t* test (two groups). *p* values < 0.05 were considered significant unless otherwise specified for multiomic network analysis.

## Results

### OLFM4 expression is upregulated in healing skin and in psoriatic lesions

**Fig. 1:**
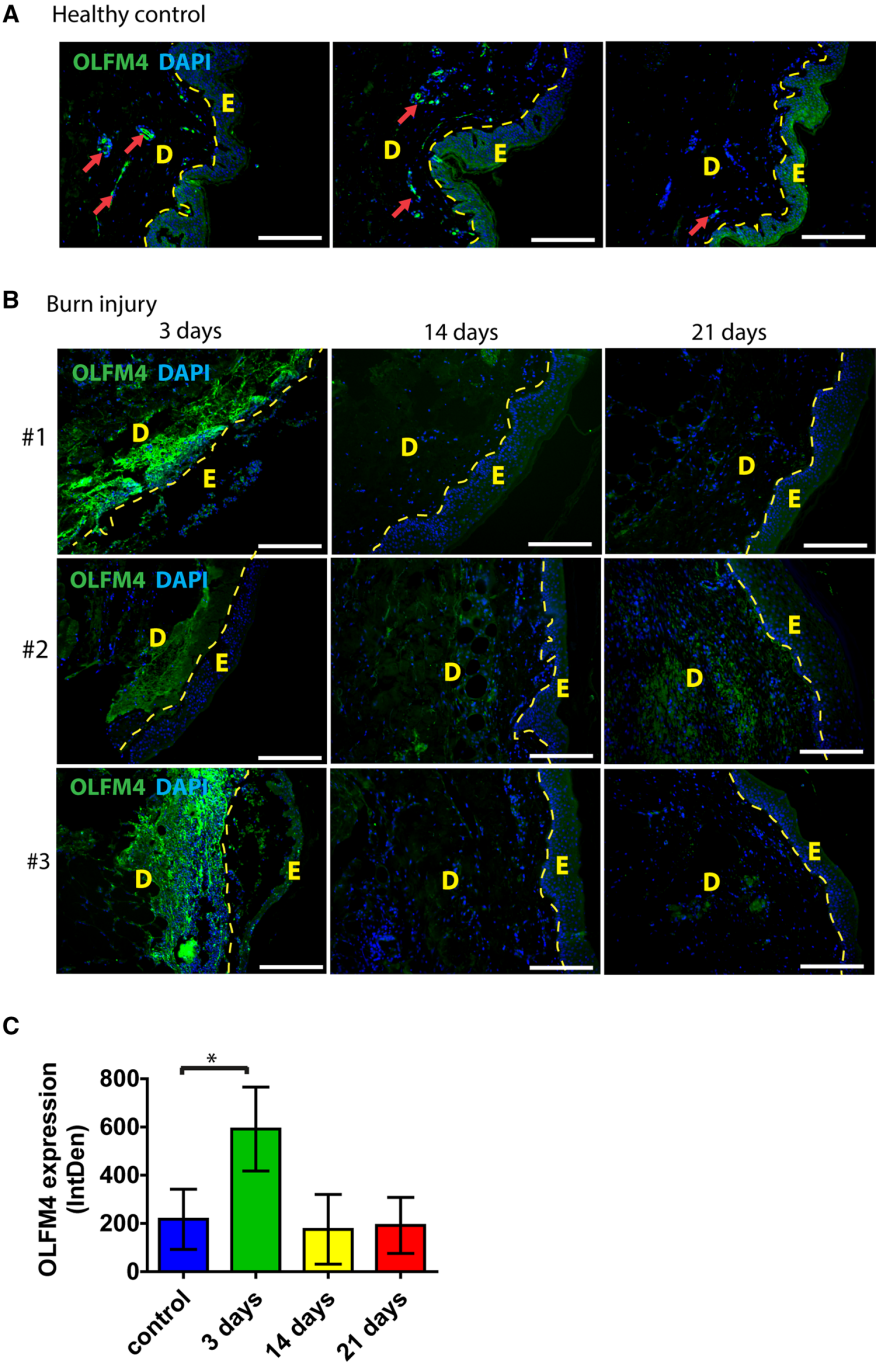
OLFM4 expression in healthy human skin and in regenerating skin after a burn injury. **(A)** Healthy control skin; **(B)** biopsy samples from the skin of burn injury patients collected at indicated timepoints after the wound excision. E—epidermis; D—dermis; OLFM4 expression in small blood vessels in dermis is indicated by red arrows. Three representative samples in each group are shown. Scale bar is 200 µm. **C** Relative quantification of OLFM4 expression by mean integrated density of the fluorescence signal. The bars depict the averages of samples from 10 patients ± standard deviation, *indicates a statistically significant (*p* < 0.05) difference

To characterize the expression of OLFM4 in healthy human skin we performed an immunofluorescence analysis of skin samples obtained during reduction mammoplasties. OLFM4 protein was detected at low levels in the epidermis and in small blood vessels of papillary dermis (Fig. 1A, Supplementary Fig. S1). In contrast, in samples of split-thickness skin graft-treated wounds from burn injury patients, high levels of OLFM4 expression were detected in healing skin areas (Fig. 1B). Strong accumulation of OLFM4 was detected in dermis under the healing wound bed at 3 days following excision and skin grafting onto the burn injury (Fig. 1B). Signal intensity quantification showed that OLFM4 signal was upregulated by 2.7-fold on average at 3 days after excision, *p* = 0.003 (Fig. 1C). However, no significant changes in OLFM4 protein levels were detected at later timepoints (14 days and 21 days), indicating that OLFM4 is transiently increased in the dermal compartment during the proliferative phase of wound healing (Fig. 1C).

We next investigated the temporal dynamics of Olfm4 expression in a mouse model of full thickness splinted wound healing. While in humans and pigs the wounds heal via the formation of granulation tissue that is later covered with epithelium (the epidermis), the rodent skin healing process includes remarkable wound contraction due to the well-developed muscular layer (*panniculus carnosus*), which is considerably less prominent in humans and pigs. The use of splinted wound model allows better comparison between human and rodent wound healing as splints minimize the contraction of rodent skin [24].

**Fig. 2:**
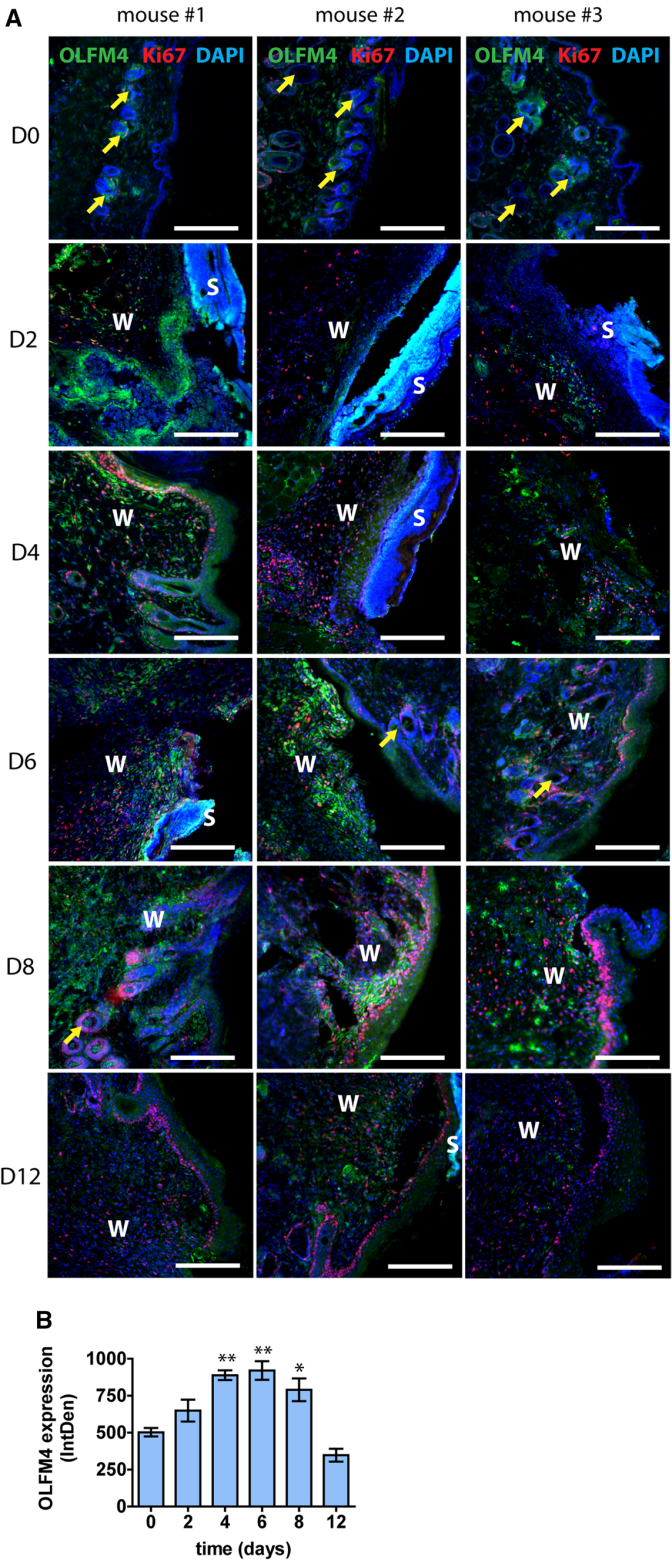
Upregulation of Olfm4 expression in regenerating mouse skin of cutaneous wounds. **A** Olfm4 expression was characterized by immunofluorescence microscopy in healthy skin (0 days) and at 2-, 4-, 6-, 8- and 12-day postwounding in a mouse model of full thickness splinted wound healing. 3 representative samples in each group are shown. Ki67 marks proliferating cells, yellow arrows indicate Olfm4 expression in hair follicles. W—wound area; S—scab. Scale bar is 200 µm. **B** Relative quantification of Olfm4 expression by mean integrated density of the fluorescence signal. Bars show the average of 3 samples for each timepoint ± standard deviation, *indicates a statistically significant (*p* < 0.05) difference from the values at day 0

In healthy mouse skin the expression of Olfm4 was detected at very low levels with most notable expression near the hair follicles and virtually no proliferating cells labelled by Ki67 were detected (Fig. 2A). Two days after wounding, we detected a minor increase in Olfm4 expression, which was accompanied with a notable increase in proliferating Ki67-positive cells. However, the strongest upregulation of Olfm4 signal was detected at days 4–6 after wounding (Fig. 2A). Quantification of the fluorescence signal showed a significant 1.8-fold upregulation of Olfm4 expression during days 4–6, *p* < 0.001 (Fig. 2B). At 8 days after injury Olfm4 expression was upregulated 1.6-fold, *p* = 0.02 which was reduced to levels comparable to day 0 (healthy control skin) at the 12-day timepoint when cell proliferation was reduced (Fig. 2A, B), indicating that similarly to the human patient samples, the expression of Olfm4 was increased after wounding and decreased thereafter.

**Fig. 3:**
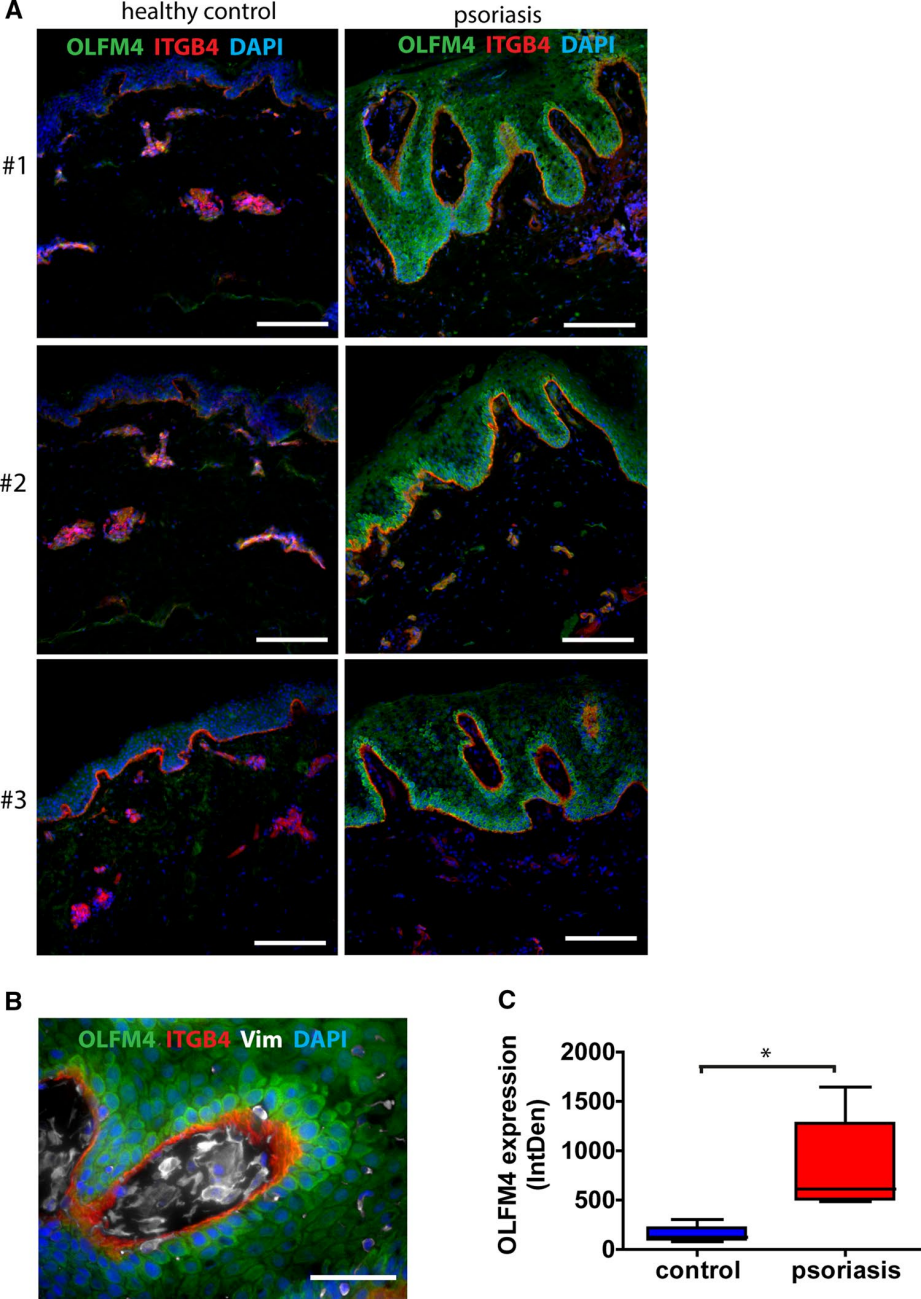
OLFM4 expression in healthy control skin and skin in psoriatic lesions. Three representative samples in each group are shown **(A)** and a higher magnification view of the OLFM4 expression in psoriatic skin lesions **(B)**. Scale bar is 200 µm on image **A** and 50 µm on image **B**. **C** Relative quantification of OLFM4 expression by mean integrated density of the fluorescence signal *n* = 5. The plot depicts the distribution of 5 samples, *indicates a statistically significant (*p* < 0.05) difference

As the histological analysis of recovering human and mouse wounds indicated that OLFM4 was induced at the proliferative phase of wound healing, we next sought to investigate whether its expression pattern was altered in skin disorders. Psoriasis is a chronic inflammatory skin disorder characterized by keratinocyte hyperproliferation [25] and is accompanied by accelerated wound healing [26]. Examination of OLFM4 expression in psoriatic lesional skin showed increased levels of the protein in epidermis compared to healthy skin (Fig. 3A, B). Quantification of the OLFM4 signal showed an average 5.3-fold upregulation in psoriatic skin samples, *p* = 0.03 (Fig. 3C). The most notable upregulation of OLFM4 expression was detected in and near the integrin beta-4 (ITGB4) expressing basal keratinocytes—the most proliferative compartment of the epidermis (Fig. 3A, B)—suggesting the presence of a link between the growth stimulating properties of OLFM4 and the hyperproliferative status of epidermis in psoriatic lesions.

### OLFM4 promotes keratinocyte but not fibroblast proliferation

**Fig. 4:**
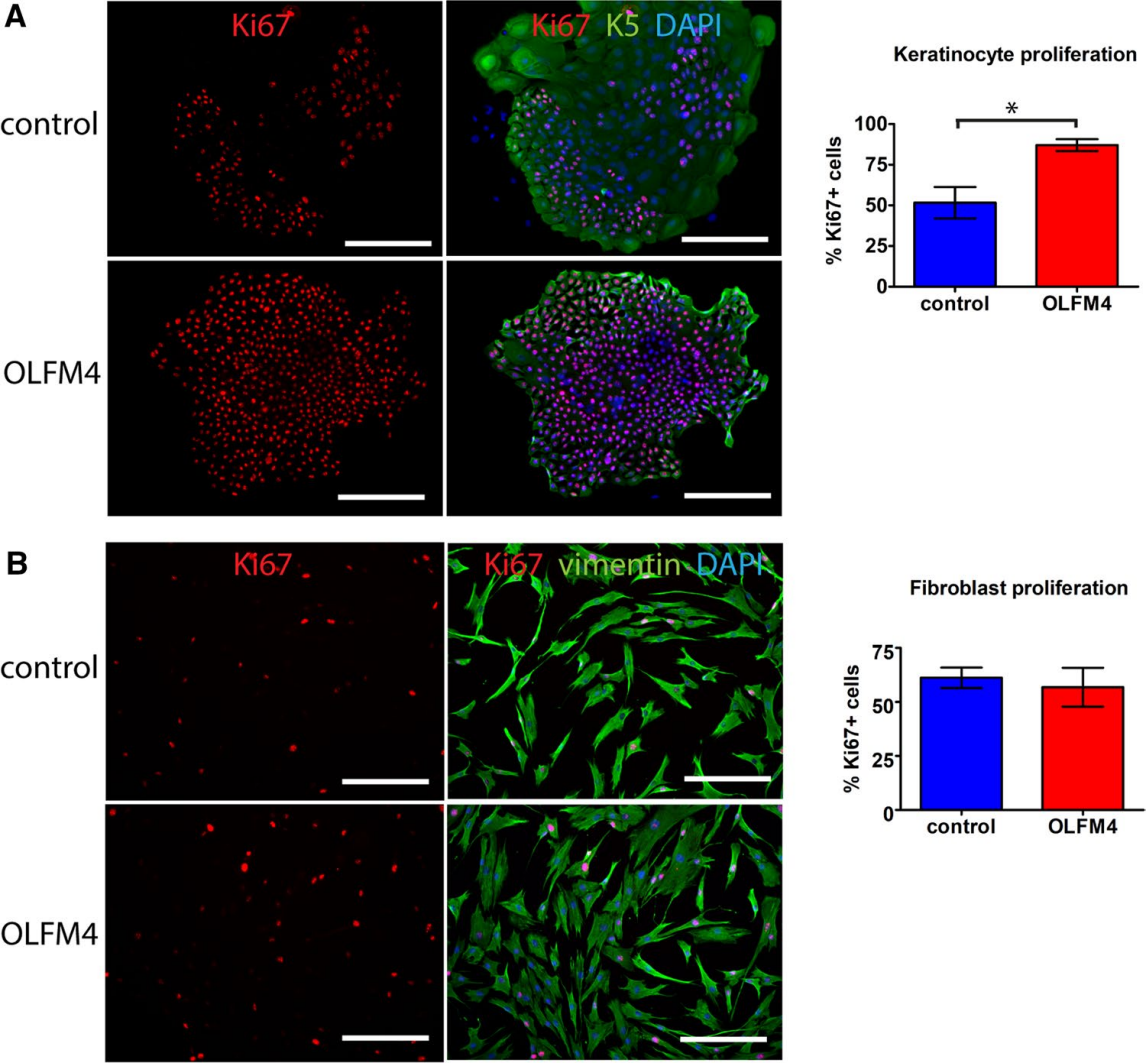
OLFM4 supports keratinocyte but not fibroblast proliferation in vitro. **A** Human primary keratinocytes were cultured in the presence of 1 µg/ml recombinant OLFM4 protein for 24 h, representative images (left) and the quantification of Ki67^+^ positive cells (right) are shown. The cytoskeleton of the cells was visualised by keratin-5 staining. **B** Human primary fibroblasts were cultured in the presence of recombinant OLFM4 protein, representative images (left) and the quantification of Ki67^+^ positive cells (right) are shown. The cytoskeleton of the cells was visualised by vimentin staining. Scale bar is 200 µm. The graphs depict the averages of at least 3 independent replicates ± standard deviation, *indicates a statistically significant (*p* < 0.05) difference compared to control cells

The data collected so far suggested a role for OLFM4 in regulating cell proliferation in the non-tumorous skin. To test the ability of OFLM4 to stimulate the proliferation of primary cells in an in vitro setting, we cultivated primary human keratinocytes for 24 h in the presence or absence of recombinant OLFM4 protein (concentration 1 µg/ml in keratinocyte growth medium) and found that the presence of external OLFM4 protein induced a 1.7-fold higher proliferation rate as characterized by Ki67 expression, *p* < 0.001 (Fig. 4A) when compared to control cells. This was accompanied by a smaller and rounder morphology of the OLFM4-stimulated cells, which is characteristic of somatic stem cells (Fig. 4A). However, no significant changes in Ki67 positivity or cell morphology were detected in fibroblasts cultured in the presence of recombinant OLFM4 protein when compared to control cells (Fig. 4B).

### Keratinocyte and fibroblast migration rate is increased in the presence of OLFM4 protein

**Fig. 5:**
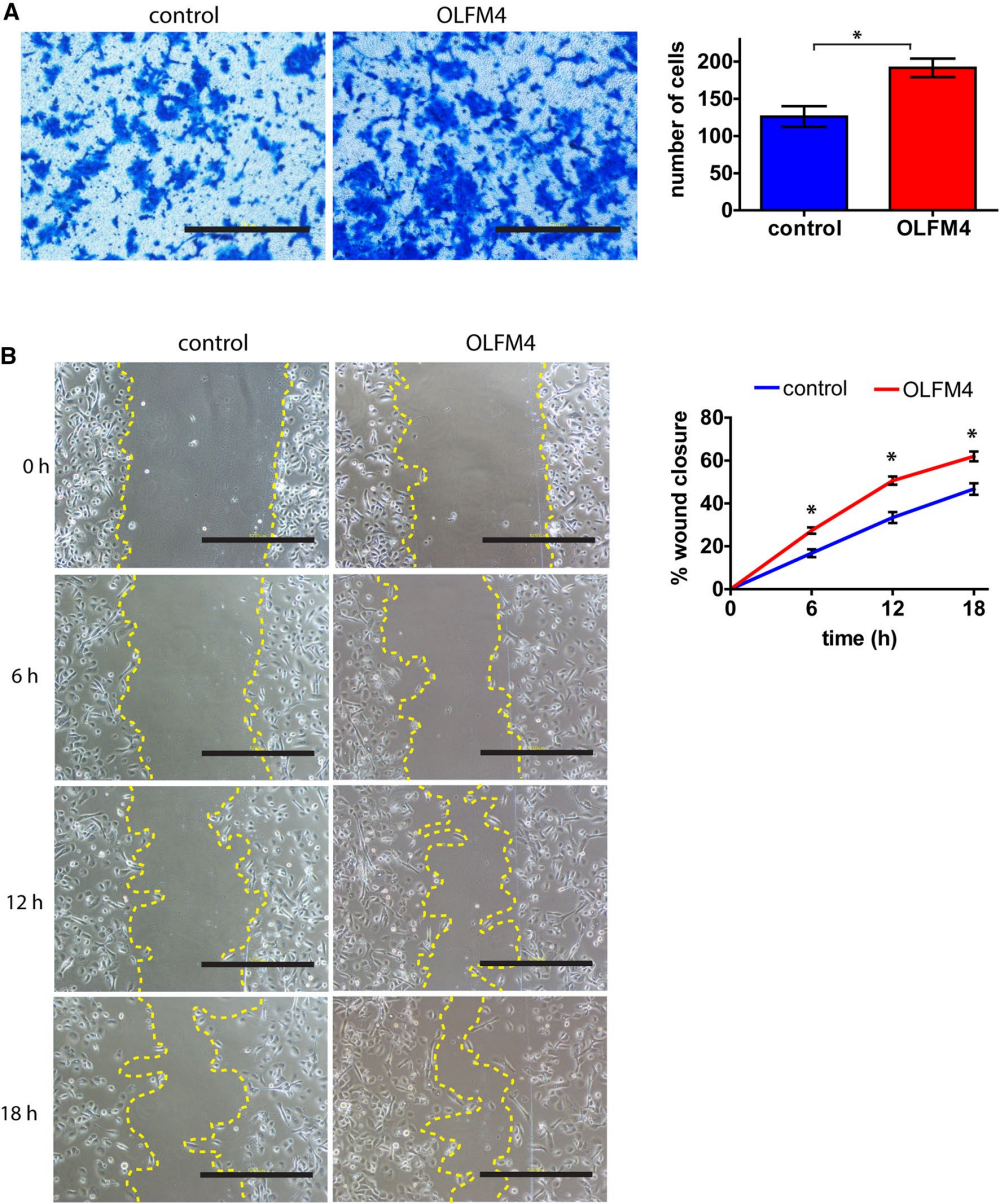
OLFM4 supports keratinocyte migration. **A** Keratinocytes were untreated (control) or stimulated for 24 h with 1 µg/ml recombinant OLFM4 protein and subsequently allowed to migrate through a transwell chamber for 24 h. Representative images (left) and the quantification of the number of migrating cells in a field of view (right) are shown. **B** In vitro scratch migration assay with keratinocytes stimulated with recombinant OLFM4, representative images at indicated timepoints (left) and the quantification of the relative wound closure in time (right) is shown. Scale bar is 200 µm. The graphs depict the averages of at least 3 independent replicates ± standard deviation, *indicates a statistically significant (*p* < 0.05) difference compared to unstimulated keratinocytes

To gain further insight into the potential mechanisms by which OLFM4 may affect cutaneous wound healing, we investigated its role in regulating cell migration. First, we conducted a Transwell migration assay that takes advantage of a plastic insert with suitably sized pores to allow cell migration from one side of the insert to the other. During the 24 h incubation, on average 1.5-fold more primary human keratinocytes stimulated with 1 µg/ml OLFM4 protein migrated across the insert when compared to control cells, *p* = 0.014 (Fig. 5A). An OLFM4-induced enhancement of cell migration rate was also evident in an in vitro scratch wound healing assay, since at the 6-h timepoint, the OLFM4-stimulated keratinocytes were able to close 27.3% of the void on average, compared 16.8% of the untreated control cells, *p* = 0.001 (Fig. 5B). The increased migration ability of OLFM4-stimulated keratinocytes was even more pronounced at the 12-h timepoint, where on average 50.6% of the wound was closed by OLFM4-stimulated cells compared to 33.4% of wound closure in the case of unstimulated control cells, *p* < 0.001 (Fig. 5B).

Similarly, the OLFM4-treated fibroblasts showed on average 1.33-fold increased Transwell migration rate compared to untreated control cells (Supplementary Fig. S2A). The differences were even more pronounced in an invasion assay using Matrigel-coated Transwell chambers, where OLFM4-stimulated fibroblasts showed 1.62-fold increased invasion rate, *p* = 0.002 (Supplementary Fig. S2B). In vitro scratch assay showed that OLFM4-treated fibroblasts were able to close the void more effectively compared to untreated control cells (Supplementary Fig. S2C) and the addition of recombinant OLFM4 protein to cell culture medium appeared to have a dose-dependent positive effect on cell migration (Supplementary Fig. S3).

### Proteotranscriptomic analysis of OLFM4-induced signalling in primary keratinocytes

To elucidate the intracellular signalling network that mediated the effects of OLFM4 stimulation in keratinocytes we analysed the proteome using LC–MS/MS and the transcriptome using RNA sequencing.

**Fig. 6:**
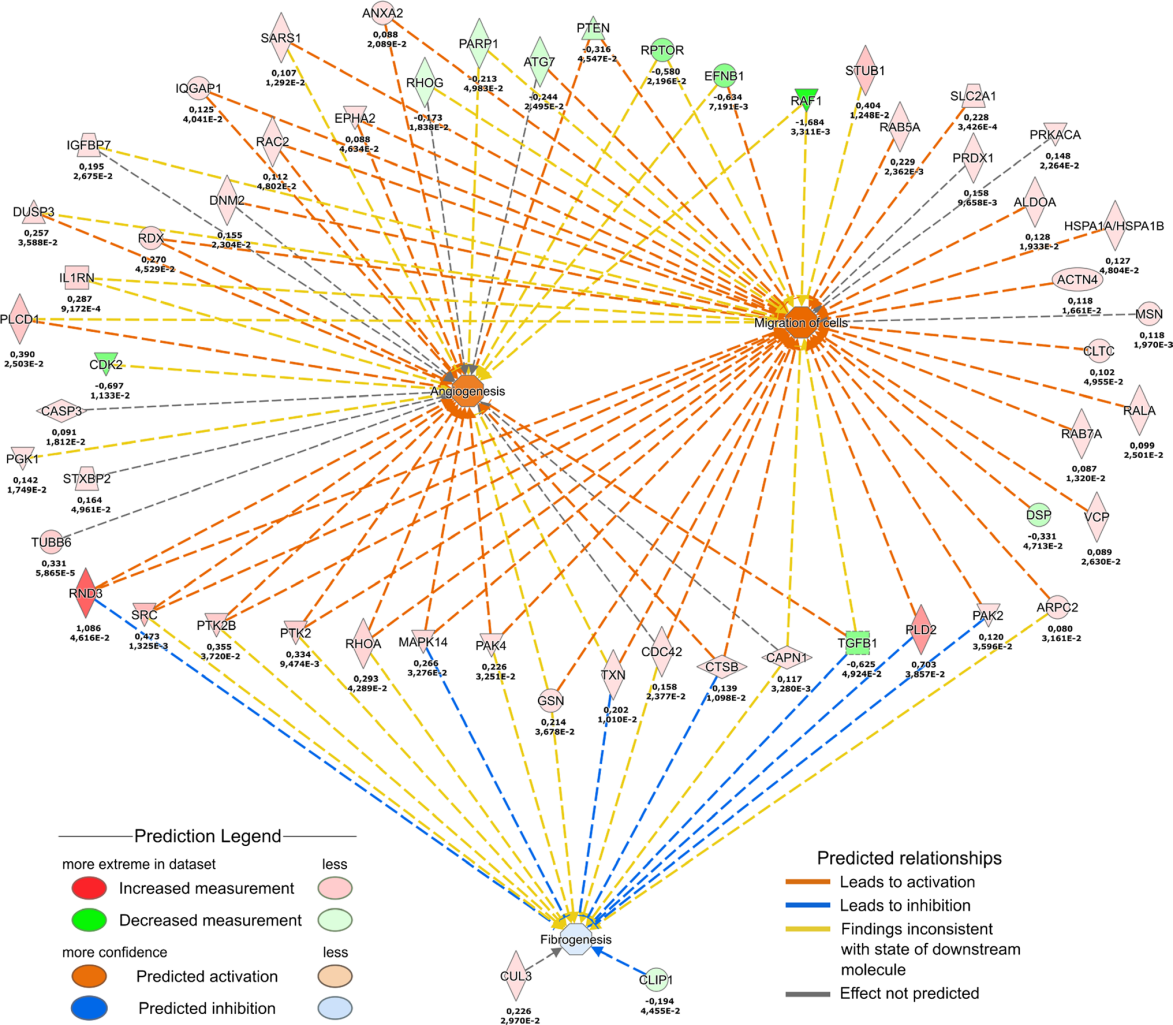
Proteomics analysis of OLFM4 stimulation in keratinocytes. Primary human keratinocytes were stimulated with 1 µg/ml recombinant OLFM4 protein for 24 h before being subjected to protein lysis and mass spectrometry analysis. The combined canonical pathway genes detected from proteomics analysis of OLFM4-stimulated keratinocytes are shown. Upper numbers indicate log2FC and lower *p* value for proteomics analysis, *n* = 3

The analysis of the protein expression profile at 24 h timepoint after stimulation with recombinant OLFM4 protein corroborated our findings (Fig. 6) as several proteins with predicted migration-promoting properties were found upregulated and some of these had well-documented roles in the regulation of migration. For example, RND3 (2.97-fold), a small GTP binding protein is involved in regulation of neuron migration [27], phospholipase D2 (PLD2; 2.02-fold) has been shown to promote phagocyte migration [28] and SRC kinase (1.60-fold) is implicated in the regulation of migration of both phagocytes and neurons among other cell types most notably via FAK kinase [29, 30]. Unexpectedly, several of the upregulated proteins were also predicted to promote angiogenesis (e.g., SRC, PTK2, PLD2, RND3; Fig. 6), a process that largely depends on cell migration. To our best knowledge, OLFM4 has not been implicated in regulation of angiogenesis before. Interestingly, a selection of upregulated proteins (RND3, MAPK14, TXN, CTSB, PLD2 and PAK2) were predicted to have anti-fibrotic effect, which may significantly contribute to the potential therapeutic effect of OLFM4 (see “Discussion”).

**Fig. 7:**
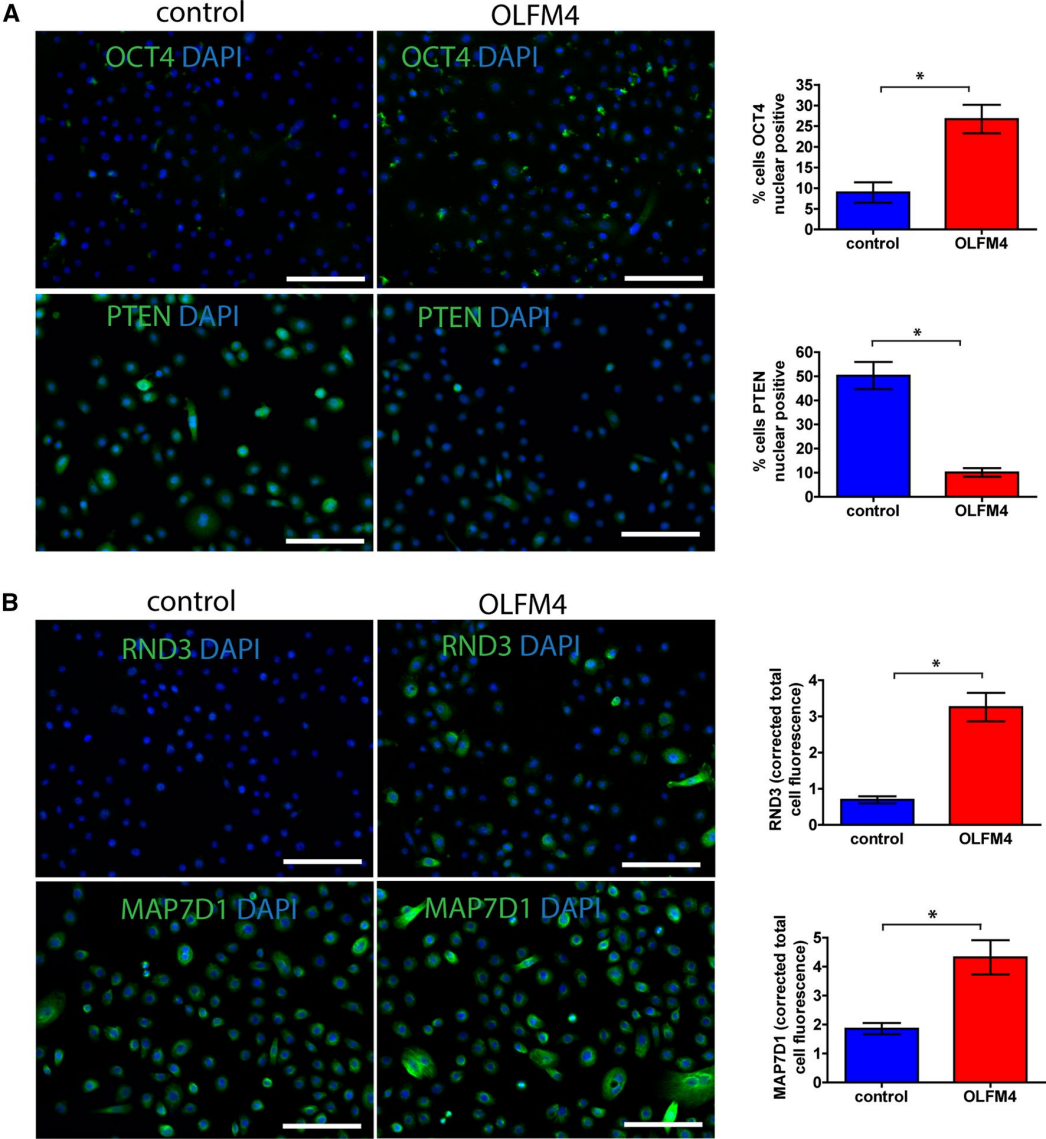
Immunofluorescence analysis of OCT4, PTEN, RND3 and MAP7D1 expression. Cultured primary keratinocytes were untreated (control) or stimulated with 1 µg/ml recombinant OLFM4 protein for 24 h. **A** Representative images of OCT4 and PTEN expression (left) and quantification of the percentage of cells expressing nuclear OCT4 and PTEN, respectively (right). **B** Representative images of RND3 and MAP7D1 expression (left) and quantification of the corrected total cell fluorescence levels (right). Scale bars are 200 µm. The graphs depict the averages of 3 replicates ± standard deviation; *indicates a statistically significant (*p* < 0.05) difference compared to unstimulated keratinocytes

Since we aimed at capturing early signalling events elicited by OLFM4 stimulation we harvested total RNA from keratinocytes that were stimulated with OLFM4 for 4 h. Transcriptome analysis demonstrated significant changes in the expression of 135 transcripts that included several transcription regulators, cytokines and growth factors (Supplementary Fig. S4, Supplementary Table S2). Pathway analysis of the OLFM4-induced changes suggested that POU5F1, ESR1 and TNF acted as central regulators of the OLFM4-induced signalling network (Supplementary Fig. S5). We detected a significant (1.83-fold) upregulation of POU5F1 encoded transcription factor OCT4 expression, which is widely associated with pluripotency and carcinogenesis [31]. The molecular pathway analysis suggested that POU5F/OCT4 was likely responsible for the downregulation of tumour suppressor protein PTEN [32] that was detected in the proteomic analysis (Fig. 6, Supplementary Fig. S4). Furthermore, the transcriptome analysis showed upregulation of several POU5F1/OCT4-regulated proteins, which were also found at increased levels at proteome level, for example BNIP2 (2.26-fold) and FECH (2.49 fold), confirming the increased activity of this transcription factor (Supplementary Fig. S5). To substantiate these findings, we analysed the expression of OCT4 and PTEN proteins in keratinocytes using immunofluorescence microscopy after 24 h culture in the presence or absence of recombinant OLFM4 protein (Fig. 7). Quantification of the fluorescence signals demonstrated 3.0-fold upregulation in the percentage of keratinocytes with OCT4 nuclear expression and 5.0-fold downregulation of the number of cells with PTEN nuclear expression (Fig. 7A).

The second major regulator of OLFM4-induced signalling, which encodes estrogen receptor α and regulates the transcription of a number of estrogen-inducible genes [33], was ESR1 (2.19-fold upregulation in transcriptomics). In line with this notion, proteomics analysis revealed that the expression of estrogen-regulated proteins INTS1, RND3, DR1, NOC3L, PXDN, ADD3, EDC3, RBM26 and MAP7D1 were increased and the expression of TOE1, MRPS5, DDX20, POLR3A, SART3, B4GALT7 were downregulated in OLFM4-treated keratinocytes (Supplementary Fig. S5). To substantiate the activation of ESR1 signalling in keratinocytes, we analysed the expression of migration- and proliferation-associated factors RND3 and MAP7D by immunofluorescence microscopy (Fig. 7B). Quantification of the fluorescence signals showed 4.7-fold upregulation of RND3 and 2.3-fold upregulation of MAP7D1 expression (Fig. 7B) indicating that more protein per cell was expressed after OLFM4 stimulation.

**Fig. 8:**
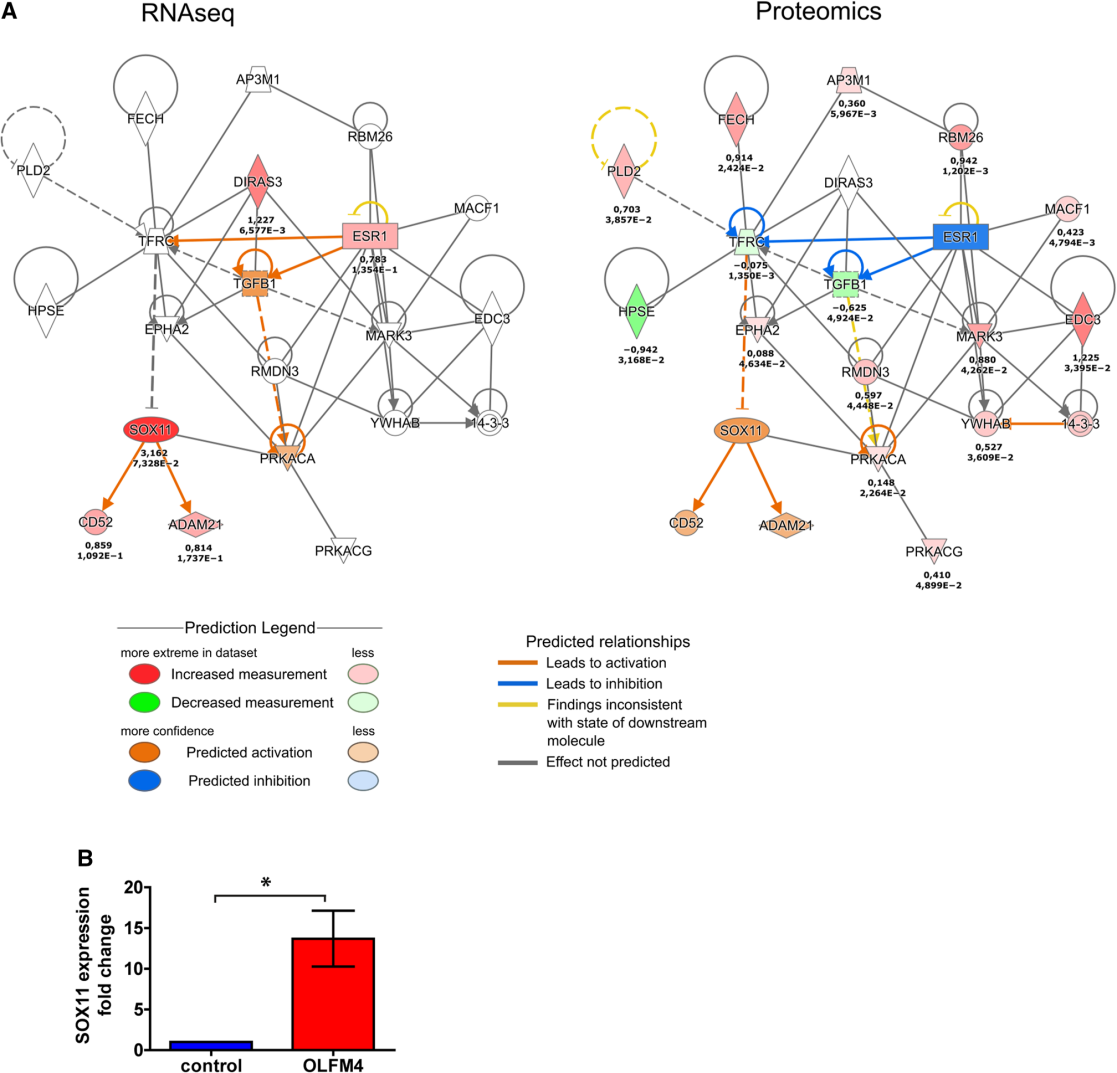
Ingenuity Pathway Analysis of ESR1, SOX11, DIRAS3 and TFRC signalling. **A** RNAseq-based signalling pathways (left) and proteomics-based pathways (right) in keratinocyte transcriptional profile in response to OLFM4 stimulation. Single numbers indicate log2FC in proteomics and duplicate values indicate log2FC and *p* value for RNAseq, *n* = 3. **B** RT-qPCR measurements of SOX11 induction after 4 h stimulation with 1 µg/ml recombinant OLFM4 protein, fold changes in mRNA expression were calculated relative to control cells. *indicates a statistically significant (*p* < 0.05) difference compared to unstimulated keratinocytes, *n* = 3

**Fig. 9:**
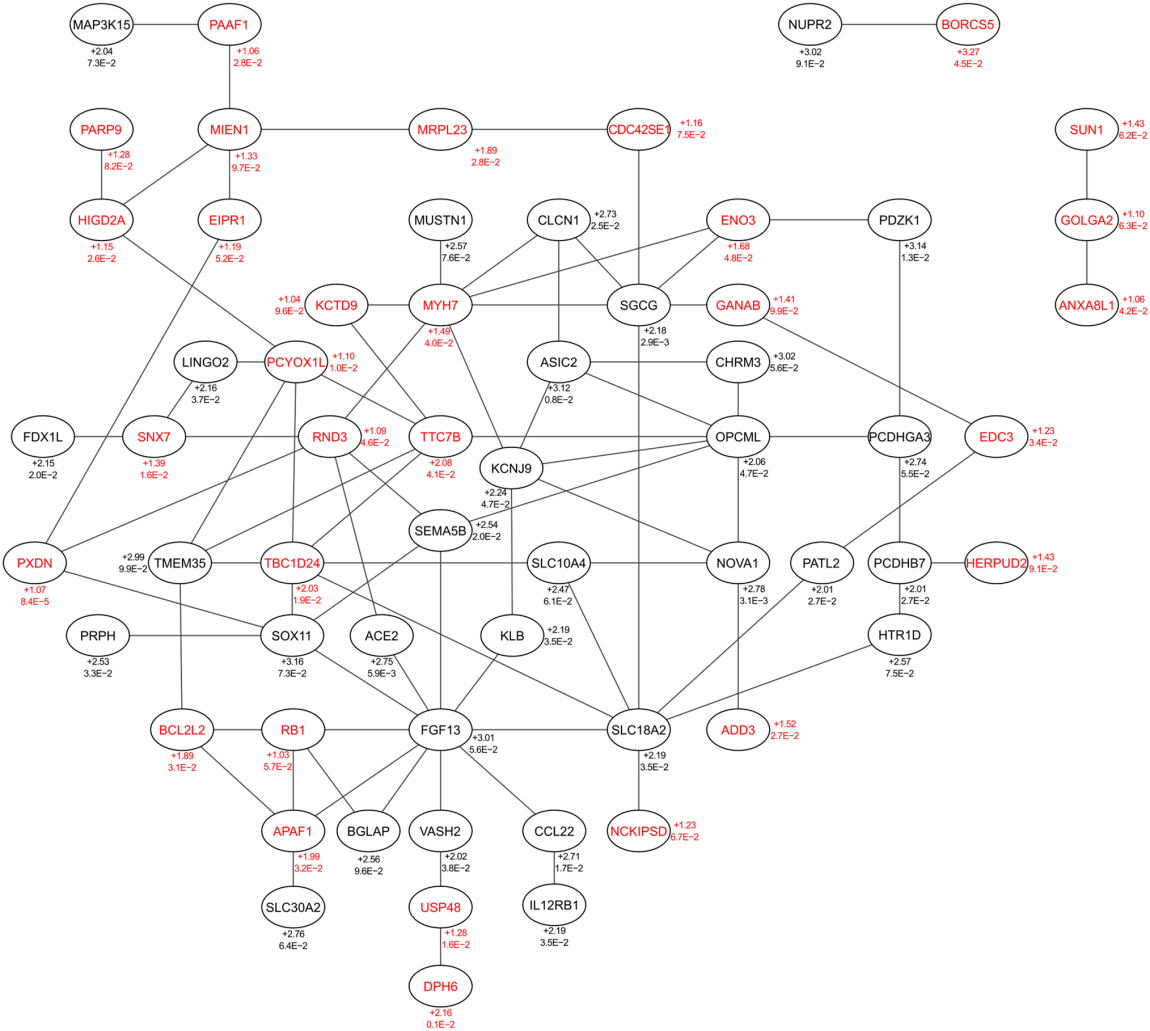
Downstream effect network of upregulated transcripts after keratinocyte stimulation with recombinant OLFM4 protein. String 11.5 network based on parameters: fold change > 2 and *p* < 0.01 in RNAseq and fold change > 1 and *p* < 0.01 in proteomics. Only upregulated DEGs (black) and DEPs (red), *n* = 81, are displayed

As ESR1 is a major ligand-activated transcription factor [33], we analysed the changes in ESR1 nuclear localization by immunofluorescence microscopy (Supplementary Fig. S6). We observed that the proportion of keratinocytes expressing nuclear ESR1 increased by 5.2-fold at 4-h stimulation with 1 µg/ml OLFM4 protein; however, at 24-h treatment ESR1 levels were reduced back to control levels (Supplementary Fig. S6). In line with this data, no changes in expression of the ESR1 at the protein level in cells treated with OLFM4 for 24 h were detected by LC–MS/MS (Fig. 8A). Transcription of *ESR1* gene is subjected to inhibitory autoregulation that was predicted to involve a network consisting of a multifunctional tumour suppressor gene DIRAS3, TGFB1, and transferrin receptor TFRC (Fig. 8A). Based on literature, one of the predicted outcomes of this feedback loop is the activation of SOX11 transcription [34]. In line with this we found a 23.6-fold upregulation of SOX11 in transcriptomic analysis of OLFM4-stimulated keratinocytes, which was confirmed by RT-qPCR corroborating the activation of the ESR1 expression (Fig. 8B). Furthermore, based on the results of both analyses, a network could be outlined that encompassed EDC3, RND4, ADD3, INTS1 at the protein level as well as ESR1, DIRAS3, SLC9A3, IL10RA and SRSF12 at the mRNA level that illustrated the activation of ESR1-coordinated signalling cascades (Supplementary Fig. S7). A proposed downstream signalling network suggested that SOX11 activation induced the expression of FGF13 and BCL2L2 that promote cell proliferation and survival (Fig. 9) [35].

### Transcriptomic analysis of OLFM4-induced signalling in primary fibroblasts

**Fig. 10:**
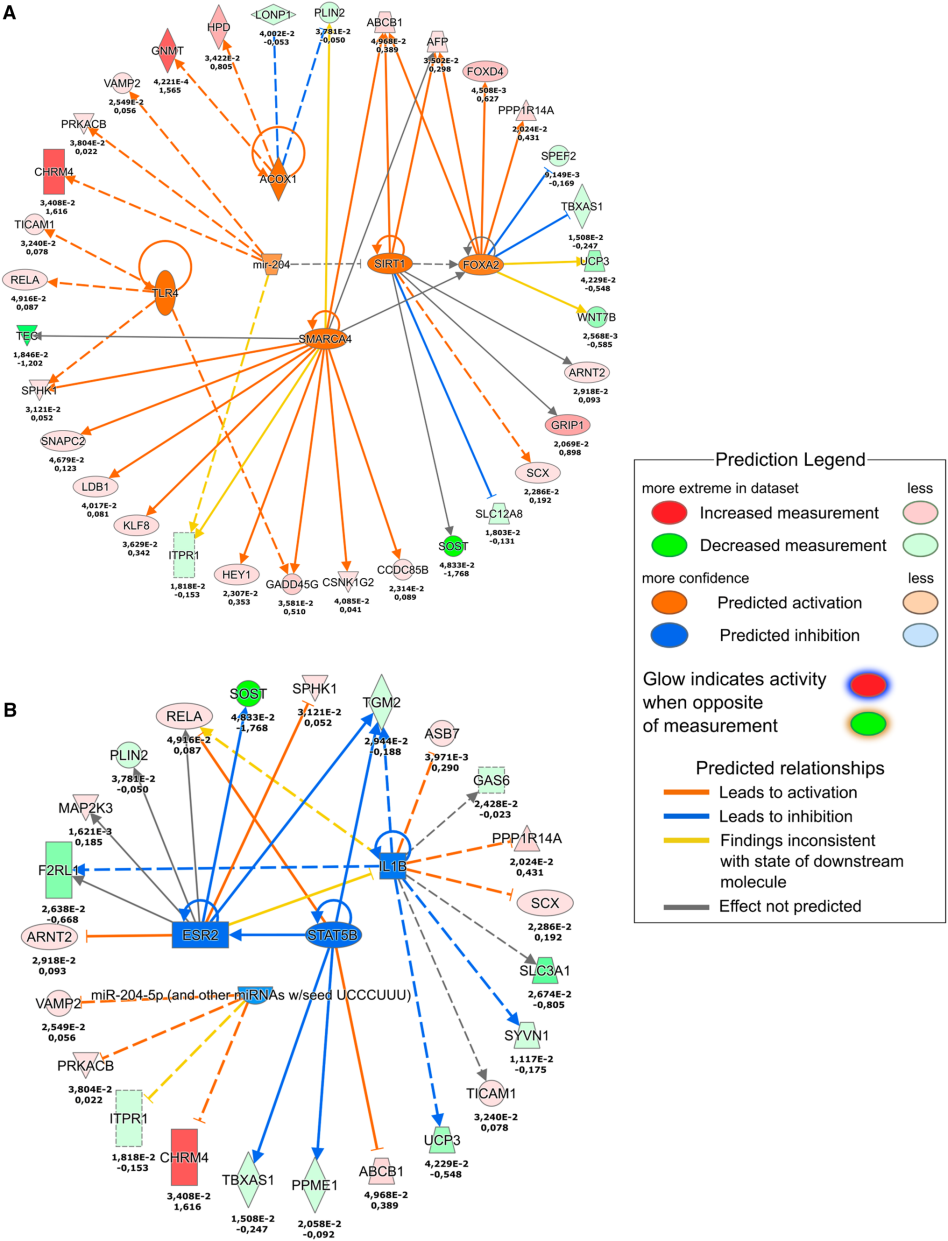
Transcriptomic analysis of OLFM4 stimulation in fibroblasts. Primary human skin fibroblasts were stimulated with 1 µg/ml recombinant OLFM4 protein for 4 h and separated RNA was analysed by RNAseq. Activated upstream signalling pathways **(A)** and inhibited upstream signalling pathways **(B)** are depicted. Upper numbers indicate the *p* value and lower log2FC for RNAseq, *n* = 2

To further elucidate the molecular mechanisms by which OLFM4 regulates fibroblast migration and invasion, we analysed the changes in the transcriptome of cultured primary fibroblasts that were stimulated for 4 h with 1 µg/ml recombinant OLFM4 protein. Ingenuity pathway analysis revealed that the predicted activation cascades were associated with signalling pathways associated with ACOX1, TLR4, mir-204, SIRT1, SMARCA4 and FOXA2 (Fig. 10A). We identified the upregulation of tumour suppressor gene GNMT (2.96-fold) and activation of GRIP1 transcription (1.86-fold). GRIP1 is essential for the formation of dermo-epidermal junctions [36]. The OLFM4-associated inhibitory signalling cascades were associated with IL1B, ESR2 and STAT5B (Fig. 10B) that indicated the downregulation of pro-inflammatory responses in fibroblasts. The most significantly downregulated transcripts were SOST (− 3.41-fold) and F2RL1 (− 1.59-fold). Subsequent functional analyses of the OLFM4-induced changes in the transcriptomes revealed activation of signalling pathways that were associated with tissue invasion of leukocytes and granulocytes as well as with the inhibition of transendothelial migration of T lymphocytes (Supplementary Fig. S8).

### Cutaneous wound healing is promoted by OLFM4 in vivo

**Fig. 11:**
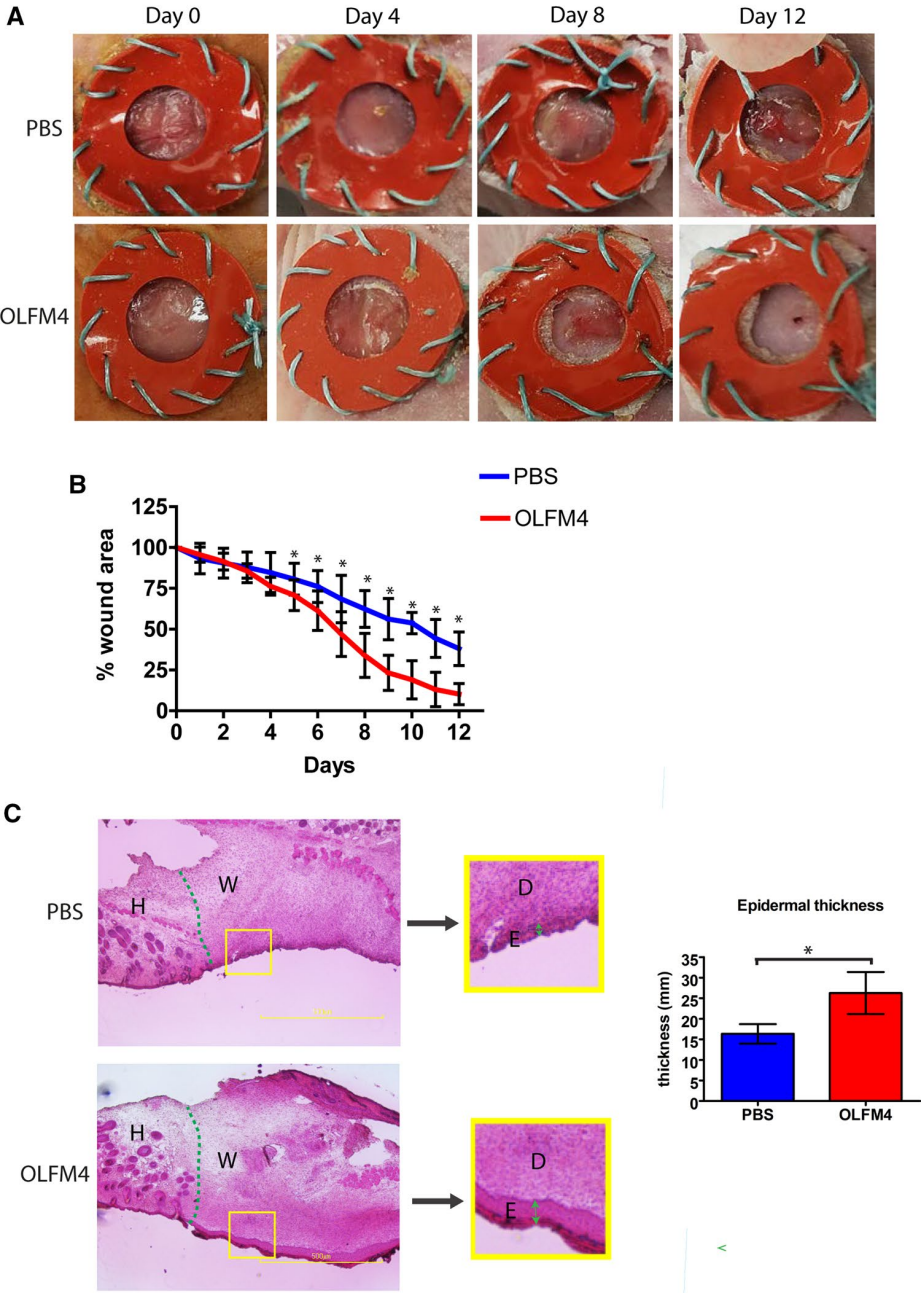
OLFM4 supports wound healing in in vivo full-thickness cutaneous wound model. Either 1 µg of recombinant OLFM4 or vehicle (PBS) was applied on inflicted dorsal wounds daily for up to 12 days. Representative images of the wounds from indicated timepoints **(A)** and measurements of the wound area **(B)**. **C** Representative histology at the time of sacrifice, hematoxylin and eosin staining. Epithelium thickness at wound edge is indicated by green arrows. H—healthy skin; W—wound; E—epidermis; D—dermis. The graphs depict the averages of 10 biological replicates ± standard deviation, *indicates a statistically significant (*p* < 0.05) difference

To further validate the anticipated role of OLFM4 in wound healing, we investigated whether the wound healing in vivo would benefit from treatment with recombinant OLFM4 protein. We applied a solution containing 1 µg of recombinant OLFM4 protein daily to full-thickness splinted dorsal wounds introduced to the BALB/c-nu/nu mice to avoid inflammatory reaction towards the recombinant protein (Fig. 11A). Compared to control (PBS-treated) wounds, OLFM4-treated wounds showed a significant reduction in wound area from 5-day postwounding when compared to control wounds (Fig. 11B). At the final 12-day timepoint, the non-healed area of the OLFM4-treated wounds was 10.3% of the initial wound size, whereas in control wounds, 38% of the initial wound was not healed suggesting a considerable positive effect of exogenous OLFM4 to the cutaneous wound healing. Histological analysis of the tissue sections at the time of sacrifice demonstrated that the OLFM4-treated wounds had thicker epidermis at the regenerating wound edge (Fig. 11C) suggesting faster re-epithelialization. Immunofluorescence analysis of basal keratinocyte marker K5 and proliferation marker Ki67 expression in the skin sections suggested that proliferative phase of wound healing had been completed in OLFM4-treated mice but was still in progress in the control group (Supplementary Fig. S9).

## Discussion

In this study we investigated for the first time the role of OLFM4 in mammalian cutaneous wound healing. We found that OLFM4 was expressed at low levels in healthy human and mouse skin. Notably, OLFM4 expression was dramatically, albeit temporarily increased in healing human skin of burn injury patients. Similarly, a strong OLFM4 expression was detected in early phases of mouse wound healing suggesting a role for this protein in skin regeneration. OLFM4 has been shown to be highly expressed in pancreatic cancer tissue, and reduction of OLFM4 mRNA expression by small interfering RNA (siRNA) inhibited cell proliferation [11], which is in accordance with our data implicating OLFM4 in regulation of cell proliferation.

Previous studies have emphasized the importance of keratinocyte migration and proliferation as well as ECM remodelling in cutaneous wound healing [1]. In our study a notable upregulation of OLFM4 expression was detected in the basal keratinocyte layer which harbours the cells with greatest proliferative capacity suggesting that this is the protein that may participate in maintaining the high regeneration potential of these cells.

A recent report has suggested that OLFM4 may have a role in the pathogenesis of generalized pustular psoriasis by activating keratinocytes via neutrophil-derived exosomes [37]. In this work we showed that OLFM4 protein was considerably upregulated in the lesional skin of plaque psoriasis patients. As our in vitro and in vivo experiments demonstrated that OLFM4 promoted keratinocyte proliferation and wound healing it is possible that this protein may participate in the pathogenesis of psoriasis by contributing to the maintenance of keratinocyte hyperproliferation in psoriatic skin lesions. Furthermore, the fact that cutaneous wounds heal faster in psoriatic skin [26] correlated well with the increased OLFM4 expression in the lesional skin areas. Moreover, molecular pathway analysis suggested that OLFM4 may mediate the reduction of tumour suppressor PTEN expression and it has been shown that PTEN expression is downregulated in psoriatic lesions [38]. PTEN expression is directly supressed by stemness factor POU5F1/OCT4 [32], which, according to our data was upregulated in OLFM4-stimulated keratinocytes. Interestingly, POU5F1 polymorphisms have been associated with psoriasis in Polish [39] and Chinese populations [40] suggesting a link between this disease and OLFM4–POU5F1 interplay.

Integrated transcriptomic and proteomic analysis suggested that OLFM4 activates ESR1 through complex regulatory mechanisms in keratinocytes. As a downstream event of activated ESR1 signalling we detected markedly increased levels of the transcription factor SOX11 that has crucial role in embryonic development but is also induced in adults upon tissue injury and cancer [41]. In mice, SOX11 activates an embryonic gene program to mediate wound repair [34] and in our experiments, the induction of cell proliferation by OLFM4 was accompanied by a smaller rounder morphology of the OLFM4-stimulated cells that is typical of multipotent somatic stem cells. Schuijers et al., (2014) showed that OLFM4 colocalized with stem cell marker LGR5 in the epithelium of small intestine [42], linking OLFM4 with stem cell identity and tissue renewal. Thus, our results suggested that by inducing SOX11 OLFM4 may activate stemness-related mechanisms in keratinocytes to facilitate wound repair. In addition, our transcriptomic and proteomic analysis also supports previously published data, which indicated that SOX11 activation induced BCL2L2 to promote cell proliferation and survival [35]. BCL2L2, a member of the BCL-2 family and one of the key regulators of apoptosis [43] is also a target of miR-29b [44]. Gu et al., 2017 showed that miR29b inhibits interferon-γ-induced keratinocyte apoptosis suggesting that BCL2L2 has an important role in inflammatory skin diseases.

Stimulation with OLFM4 increased the migration rate of human primary keratinocytes as well as fibroblasts. Our results are in agreement with previous findings that demonstrated a role for OLFM4 in promoting tumour cell adhesion and migration [9]. We detected activation of SMARCA4, a chromatin binding protein that is associated with tumorigenesis in a variety of tissues [45]. As SMARCA4 expression is negatively associated with CD8 + T cell infiltration of tumours [45], it has been suggested to control the magnitude and duration of inflammation. In addition, OLFM4 stimulation was predicted to activate TLR4 signalling that is an important regulator of inflammation during cutaneous wound healing [46, 47]. Moreover, OLFM4 inhibited IL1B signalling in fibroblasts, which could enhance the wound healing process, as previous studies have shown that blocking IL1B can improve wound healing in diabetic mice [48].

The current manuscript, however, has several limitations. Due to our limited capacity to perform wound healing experiments only a single concentration of OLFM4 was used and the amount of administered OLFM4 was considerably exceeding the anticipated physiological levels of this protein leaving the role of physiological levels of this protein open. Moreover, it could be expected that cells responsive to OLFM4 differ in their early and delayed molecular level signalling patterns to OLFM4 stimulus. This, in turn would lead to the alterations in cell behaviour over time. Thus, choosing just one timepoint for pathway analysis may potentially give a restricted and incomplete picture of the signalling cascades induced by OLFM4.

Taken together, we showed through in vitro and in vivo experiments that OLFM4 supported wound healing by stimulating keratinocyte proliferation and increasing both keratinocyte and fibroblast migration. Our proteotranscriptomic analysis suggested the involvement of OLFM4 in activating stem/progenitor cell programs and regulating inflammatory response. Thus, one can envision, that incorporation of OLFM4 into wound treatment modalities could enhance their therapeutic potential. The next steps in this regard would include an in-depth analysis of signalling cascades regulated by OLFM4 at multiple timepoints, elucidation of the role of cell-autonomous OLFM4 signalling and the role of OLFM4 in regulation of various skin functions using either in vivo gene silencing or conditional OLFM4 knock-out mice. An interesting aspect worth of further studies is also the potential involvement of OLFM4 in the pathogenesis of psoriasis and prevention of fibrosis. Further information on the potency of OLFM4 to contribute to the treatment of hard-to-heal wounds can be gained by testing OLFM4 in chronic wounds in in vivo models and by assessing possible ways to incorporate OLFM4 in existing wound dressings.

### Supplementary Information

Below is the link to the electronic supplementary material.

Supplementary file1 (DOCX 14 KB)

Supplementary file2 (XLSX 47 KB)

Supplementary file3 (TIF 758 KB)

Supplementary file4 (TIF 6075 KB)

Supplementary file5 (TIF 4962 KB)

Supplementary file6 (TIF 6578 KB)

Supplementary file7 (TIF 2640 KB)

Supplementary file8 (TIF 1966 KB)

Supplementary file9 (TIF 378 KB)

Supplementary file10 (TIF 796 KB)

Supplementary file11 (TIF 4452 KB)

## Data Availability

The RNA sequencing data were uploaded to Gene Expression Omnibus (https://www.ncbi.nlm.nih.gov/geo/) (GEO accession no GSE188919). The proteomics data are available via ProteomeXchange with identifier PXD029881.
