# Supplementary material for: Olfactomedin-4 improves cutaneous wound healing by promoting skin cell proliferation and migration through POU5F1/OCT4 and ESR1 signalling cascades
Source: Cell Mol Life Sci. 2022 Feb 26;79(3):157. doi: 10.1007/s00018-022-04202-8 (PMC8882121; doi:10.1007/s00018-022-04202-8)
Supplement: Supplementary file 1 — Supplementary file1 (DOCX 14 KB) [file 18_2022_4202_MOESM1_ESM.docx]

**Supplementary Table 1.** Antibodies used in immunofluorescence analysis.

| **Antibody** | **Host, cat no** | **Dilution** | **Source** |
| --- | --- | --- | --- |
| Alexa Flour 488 Donkey anti-Goat | A11055 | 1:1000 | Thermo Fisher Scientific (Eugene, OR, USA) |
| Alexa Flour 488 Donkey anti-Rabbit | A21207 | 1:1000 | Thermo Fisher Scientific (Eugene, OR, USA) |
| Alexa Flour 568 Donkey anti-Rabbit | A10042 | 1:1000 | Thermo Fisher Scientific (Eugene, OR, USA) |
| Alexa Flour 647 Donkey anti-Mouse | A31571 | 1:1000 | Thermo Fisher Scientific (Eugene, OR, USA) |
| αSMA | NCL-SMA | 1:50 | Leica Biosystems (Wetzlar, Germany) |
| ESR1 | Rabbit, HPA000450 | 1:200 | Atlas Antibodies (Bromma, Sweden) |
| Integrin beta 4 | Mouse, FAB4060R-100UG | 1:200 | R&D Systems (Minneapolis, MN, USA) |
| Keratin-5 | Rabbit, ab53121 | 1:200 | Abcam (Cambridge, UK) |
| Ki67 | Rat, 14-5698 | 1:200 | eBioscience (San Diego, CA, USA) |
| MAP7D1 | Rabbit, HPA028075 | 1:50 | Atlas Antibodies (Bromma, Sweden) |
| OCT3/4 | Goat, sc-5279 | 1:50 | Santa Cruz Biotechnology (Dallas, TX, USA) |
| OLFM4 (anti-human) | Rabbit, NBP2-24535SS | 1:100 | Novus Biologicals (Abingdon, UK) |
| OLFM4 (anti-mouse) | Rabbit, PAA162Mu01 | 1:100 | Cloud-Clone Corp. |
| PTEN | Rabbit, #9552 | 1:100 | Cell Signaling Technology (Danvers, MA, USA) |
| RND3 | Rabbit, HPA060504 | 1:100 | Atlas Antibodies (Bromma, Sweden) |
| Vimentin | Rabbit, ab92547 | 1:250 | Abcam (Cambridge, UK) |
