# Supplementary figures and images for: Olfactomedin-4 improves cutaneous wound healing by promoting skin cell proliferation and migration through POU5F1/OCT4 and ESR1 signalling cascades
Source: Cell Mol Life Sci. 2022 Feb 26;79(3):157. doi: 10.1007/s00018-022-04202-8 (PMC8882121; doi:10.1007/s00018-022-04202-8)

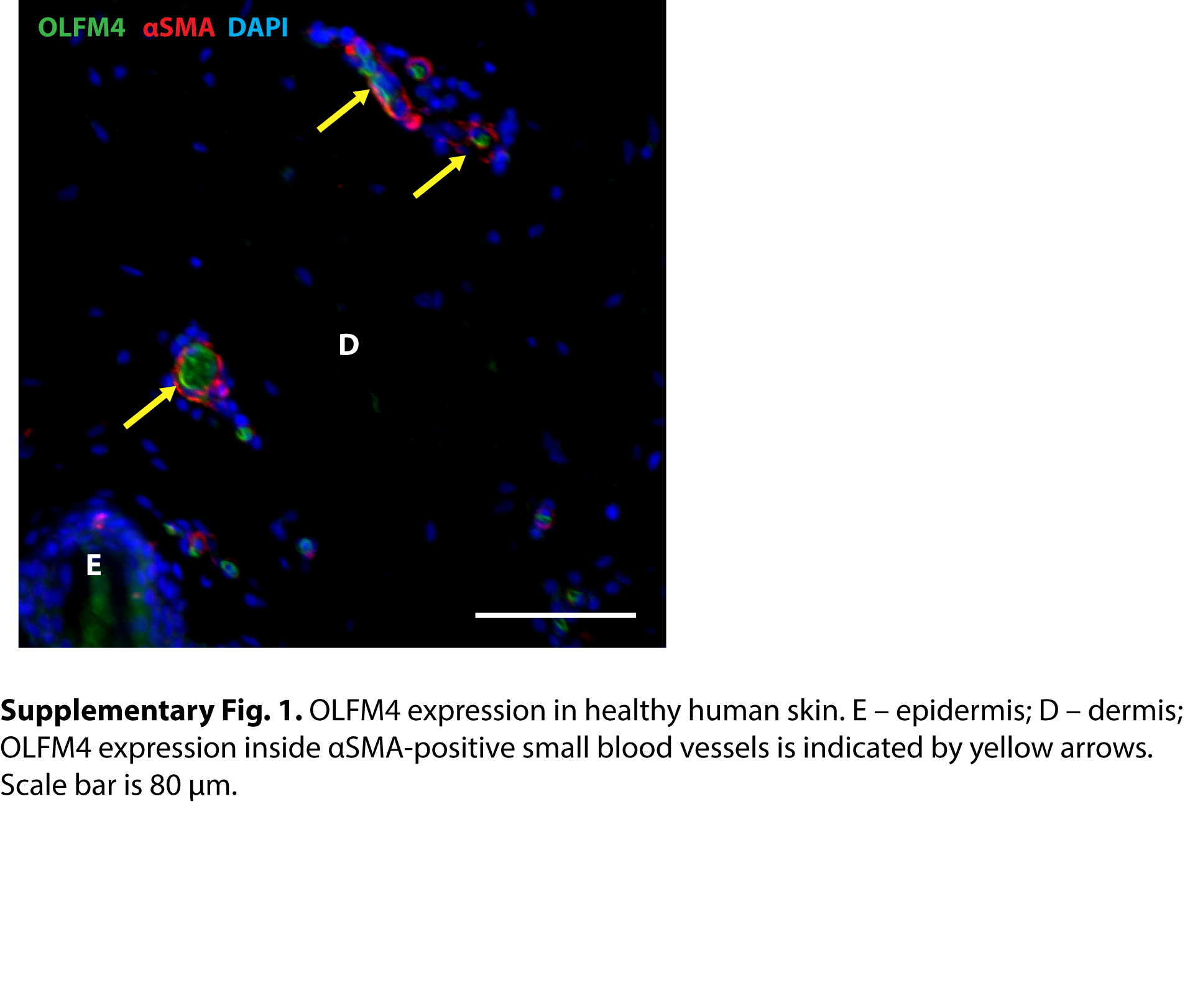

Supplement: Supplementary file 3 — Supplementary file3 (TIF 758 KB) [file 18_2022_4202_MOESM3_ESM.tif]

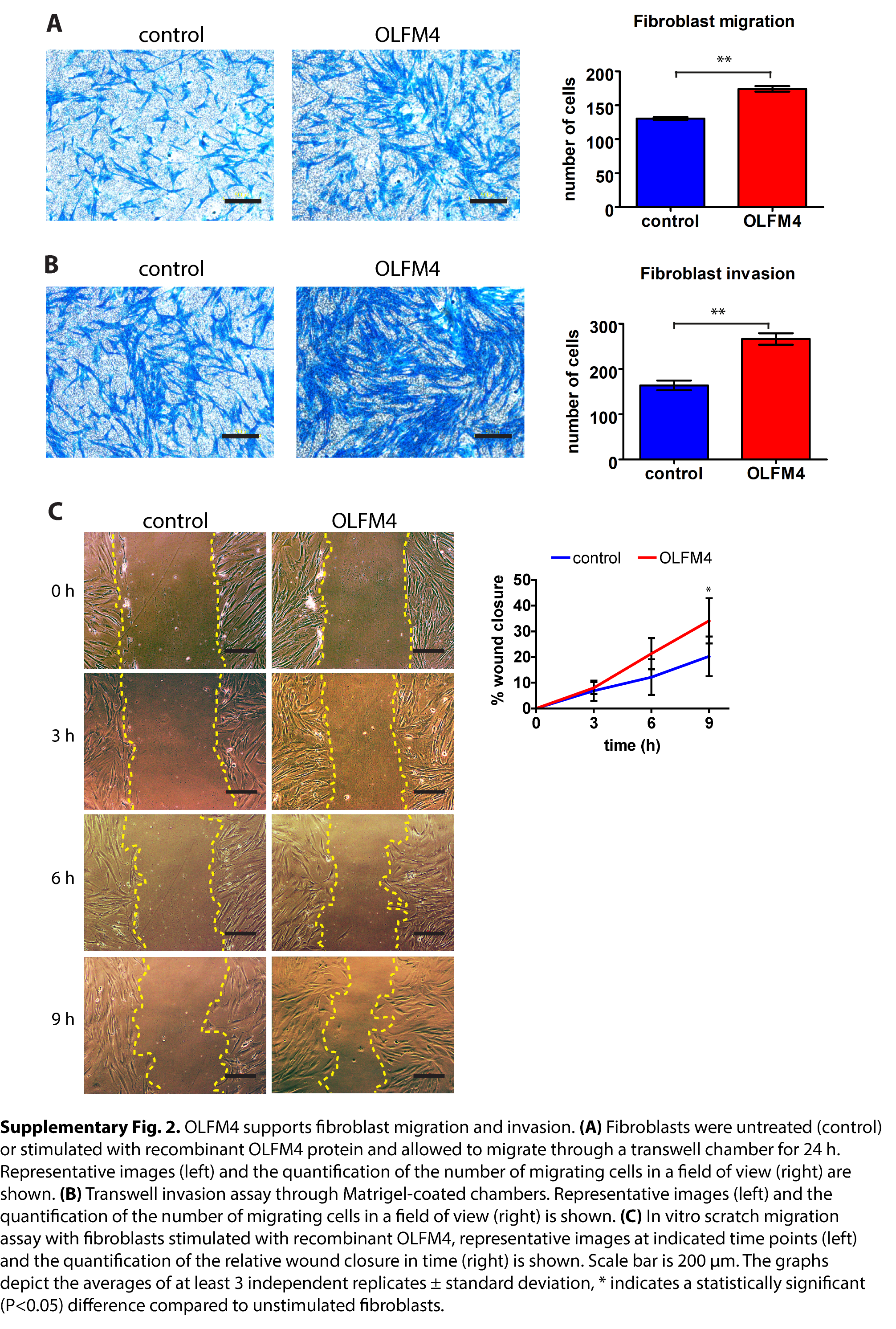

Supplement: Supplementary file 4 — Supplementary file4 (TIF 6075 KB) [file 18_2022_4202_MOESM4_ESM.tif]

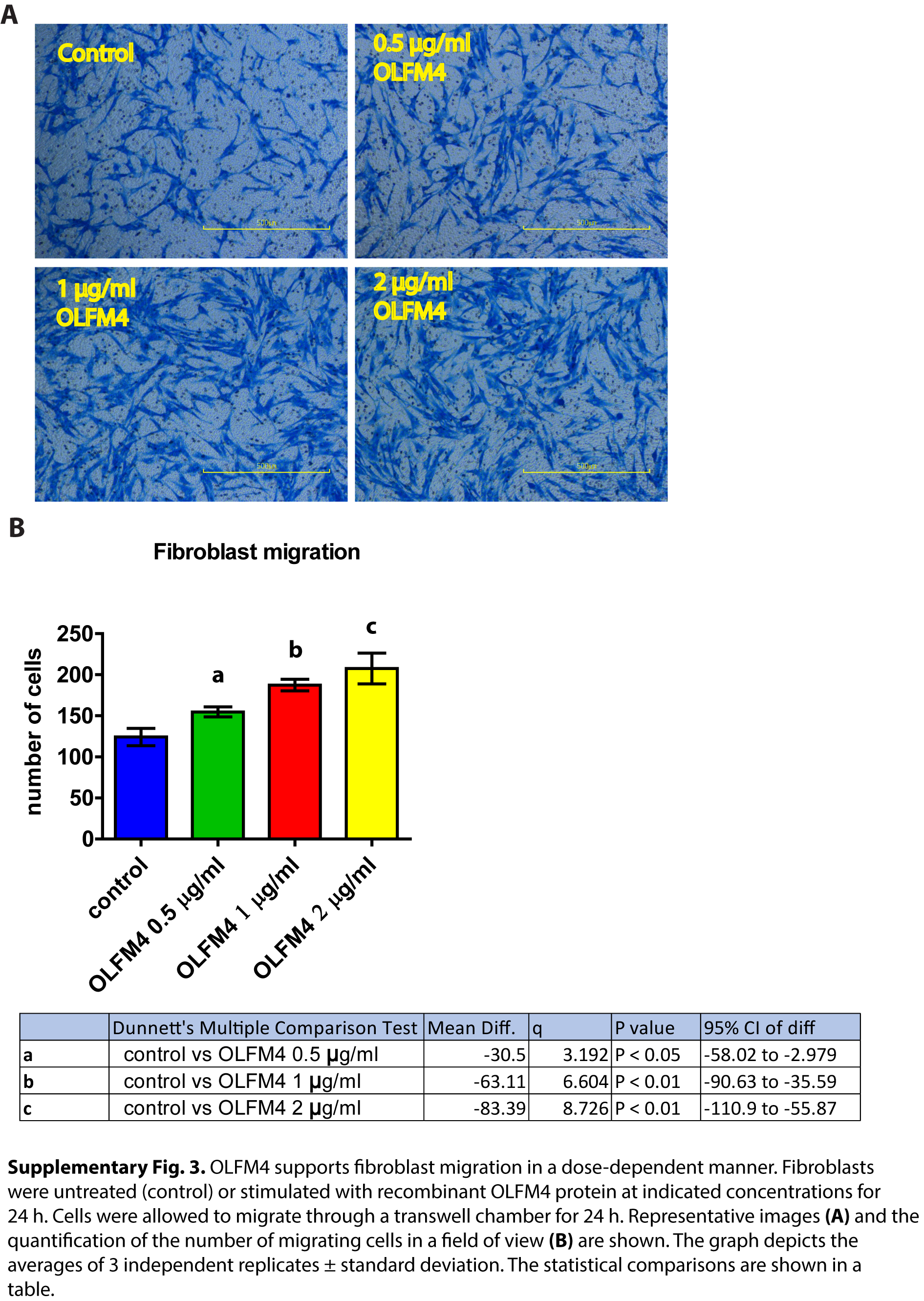

Supplement: Supplementary file 5 — Supplementary file5 (TIF 4962 KB) [file 18_2022_4202_MOESM5_ESM.tif]

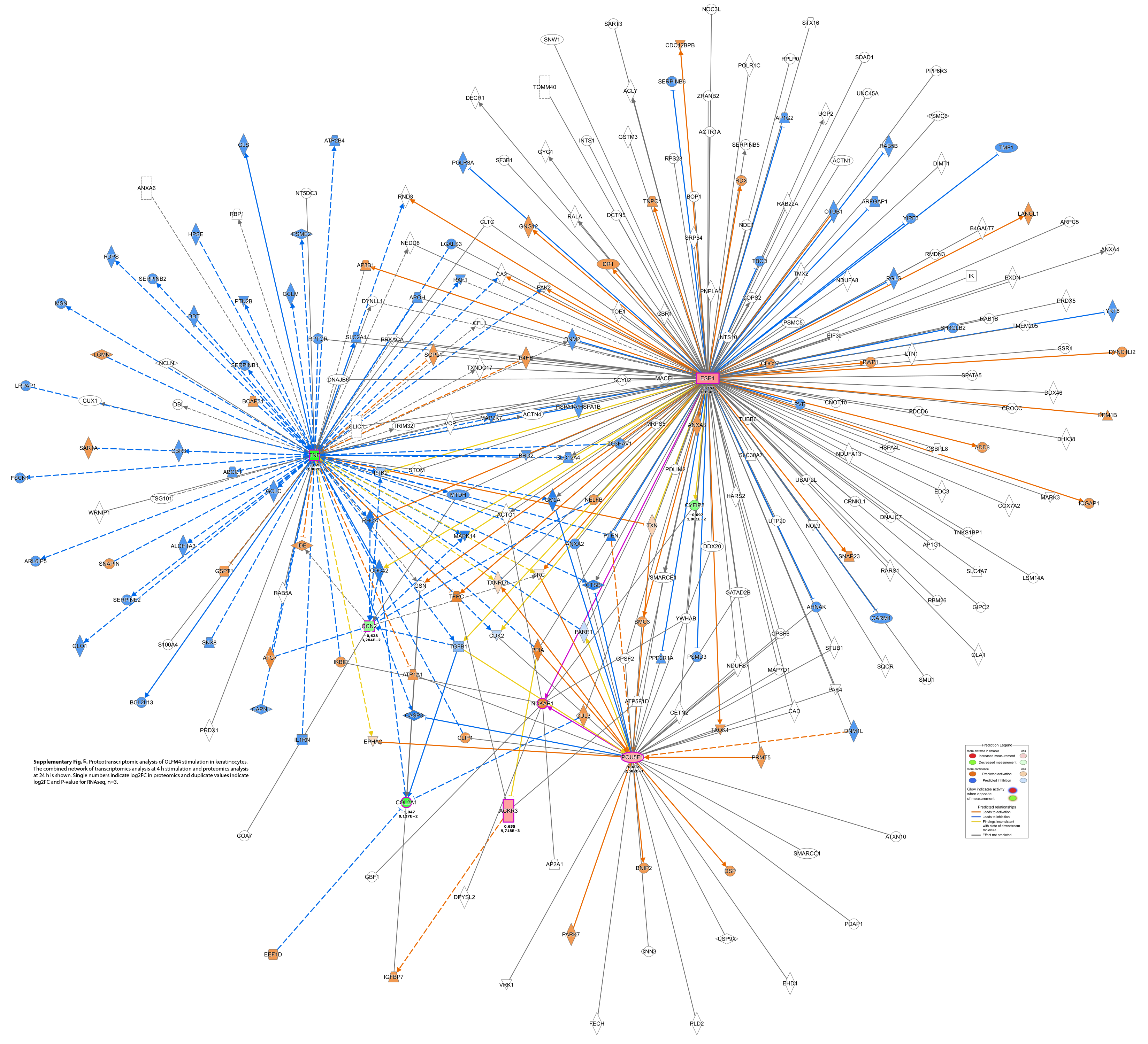

Supplement: Supplementary file 7 — Supplementary file7 (TIF 2640 KB) [file 18_2022_4202_MOESM7_ESM.tif]

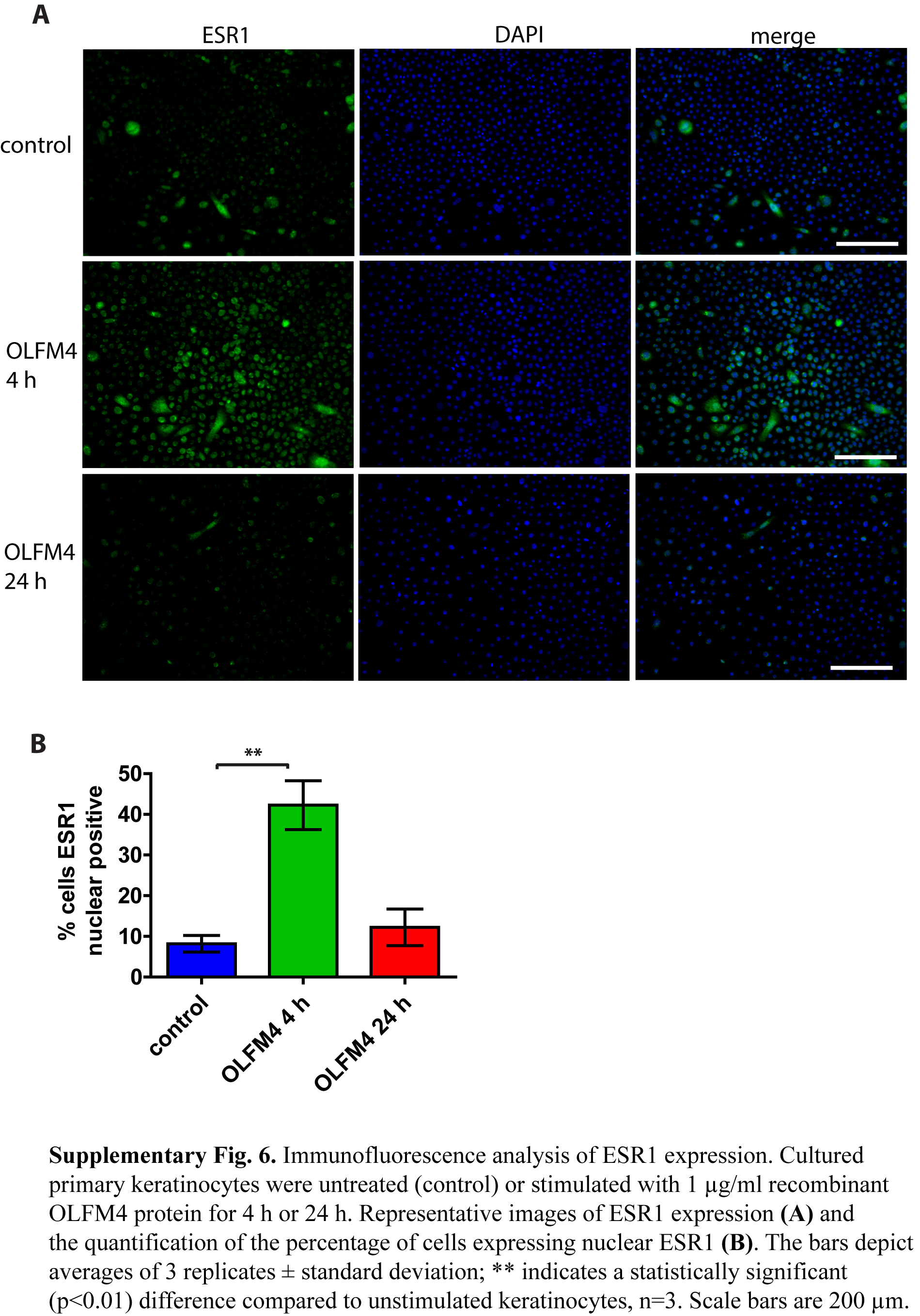

Supplement: Supplementary file 8 — Supplementary file8 (TIF 1966 KB) [file 18_2022_4202_MOESM8_ESM.tif]

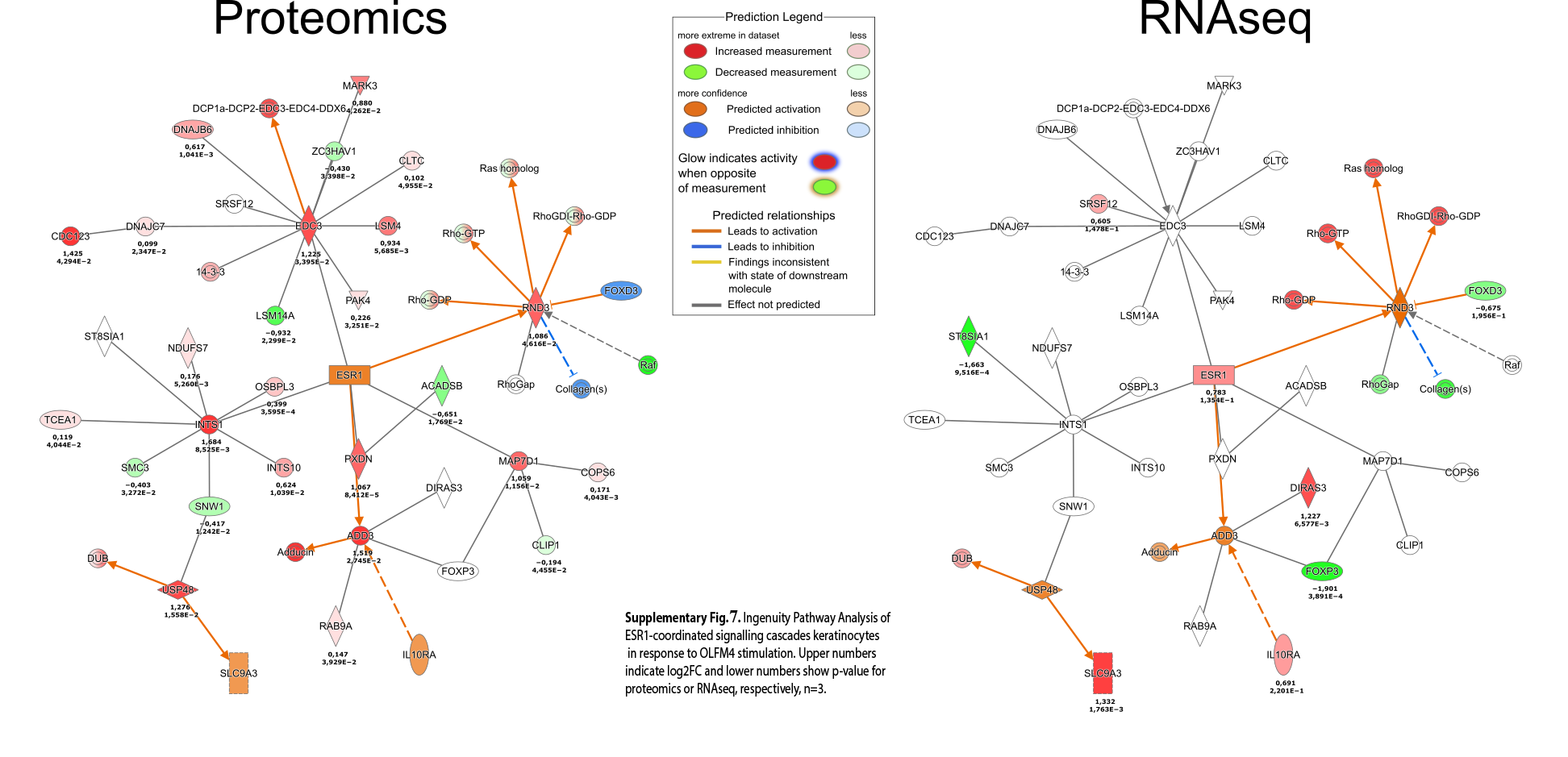

Supplement: Supplementary file 9 — Supplementary file9 (TIF 378 KB) [file 18_2022_4202_MOESM9_ESM.tif]

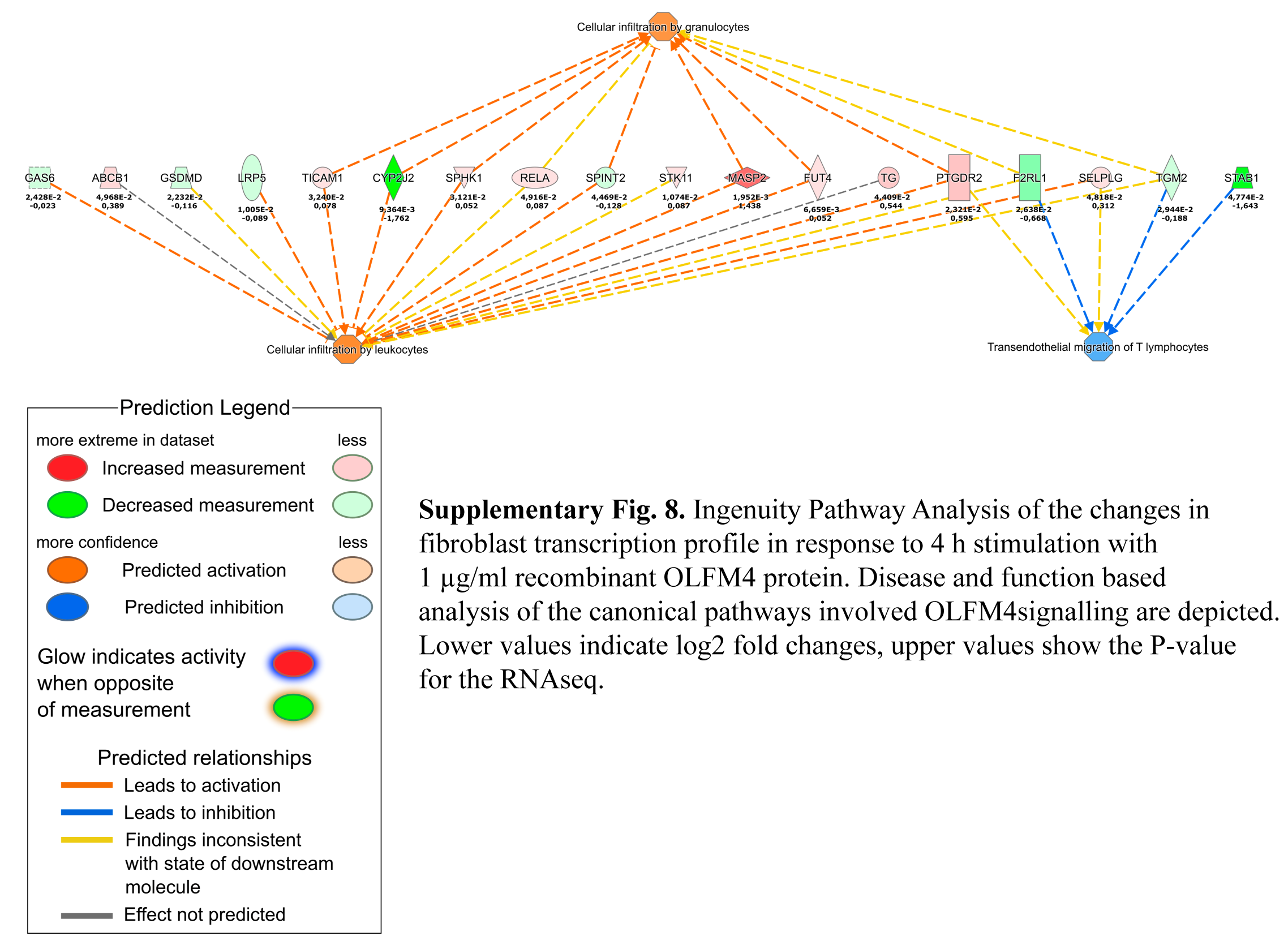

Supplement: Supplementary file 10 — Supplementary file10 (TIF 796 KB) [file 18_2022_4202_MOESM10_ESM.tif]

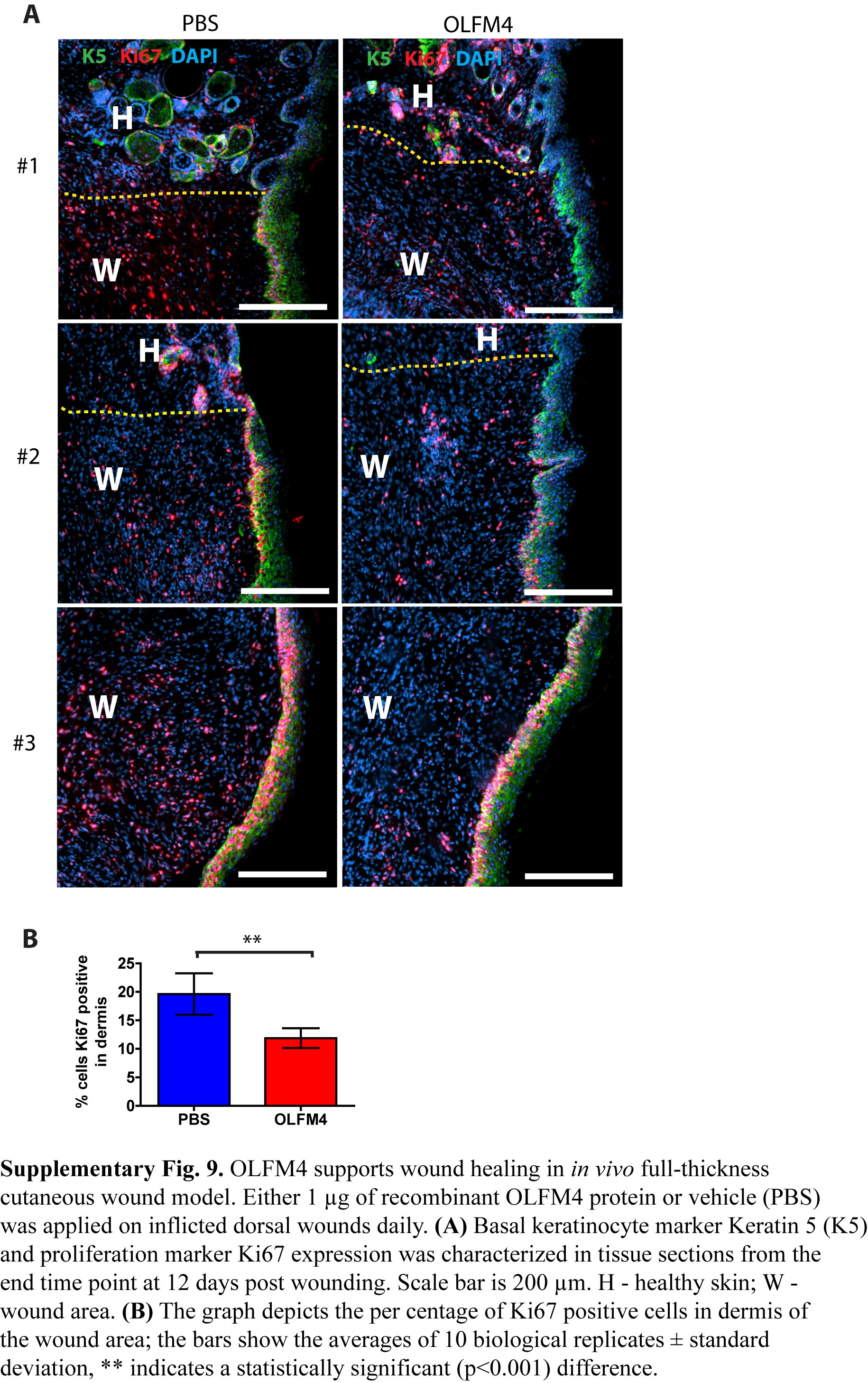

Supplement: Supplementary file 11 — Supplementary file11 (TIF 4452 KB) [file 18_2022_4202_MOESM11_ESM.tif]
